# Predicting Autism Spectrum Disorder in Children Using Glowworm Optimization With Extreme Learning Machine Networks

**DOI:** 10.1002/brb3.71225

**Published:** 2026-02-16

**Authors:** Vijay Govindarajan, Ashit Kumar Dutta, Zaffar Ahmed Shaikh, Amr Yousef, Mohd Anjum, Sana Shahab

**Affiliations:** ^1^ Distribution and Supply Technology Expedia Group Seattle Washington USA; ^2^ Department of Computer Science and Information Systems College of Applied Sciences AlMaarefa University, Dariyah Riyadh Saudi Arabia; ^3^ Research Center, Deanship of Scientific Research and Post‐Graduate Studies AlMaarefa University, Dariyah Riyadh Saudi Arabia; ^4^ Department of Computer Science and Information Technology Benazir Bhutto Shaheed University Lyari Karachi Pakistan; ^5^ School of Engineering École Polytechnique Fédérale de Lausanne Lausanne Switzerland; ^6^ Electrical Engineering Department University of Business and Technology Jeddah Saudi Arabia; ^7^ Engineering Mathematics Department Alexandria University Alexandria Egypt; ^8^ Department of Computer Engineering Aligarh Muslim University Aligarh India; ^9^ Department of Business Administration College of Business Administration Princess Nourah Bint Abdulrahman University Riyadh Saudi Arabia

**Keywords:** autism spectrum disorder, glowworm optimization with extreme learning machine networks, hyperparameters, pediatric healthcare

## Abstract

**Purpose:**

The earlier prediction of autism spectrum disorder (ASD) placed a serious attention on ensuring the appropriate intervention to improve the child's behavioral, cognitive, and social development. The previous detection process is commonly time‐intensive, subjective, and highly dependent on the clinical professions, which leads to limited accessibility in rural areas. The difficulties are addressed by introducing effective ASD detection systems, which provide a scalable, objective, and fast solution, reducing the challenges in the healthcare environment.

**Method:**

This work integrates the Glowworm Optimization with Extreme Learning Machine Networks (GO‐ELMN) model to enhance the efficiency of ASD prediction. During the analysis, ASD screening data for children are collected and processed frequently to obtain behavioral, demographic, and medical features. The extracted features are processed by an extraction learning technique, in which the network hyperparameters are optimized using the glowworm optimization algorithm. The optimized classifier recognizes children's behavior by addressing the issues of limited and imbalanced data.

**Findings:**

The efficiency of the system is evaluated using experimental results, in which the system ensures high accuracy and convergence speed.

**Conclusion:**

The ASD detection model provides an interpretable, fast, and reliable solution that is effectively utilized in the pediatric healthcare domain.

## Graphical abstract

The proposed framework for ASD detection has been illustrated. The process begins with dataset collection and preprocessing, followed by feature selection and hyperparameter optimization using GSO. The optimized features are classified through an ELM classifier, yielding high accuracy, fast convergence, and low computational cost for reliable ASD prediction.

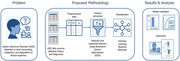

## 1 Introduction

Autism spectrum disorder (ASD) is a lifelong condition associated with a person's neurological development concerning perceiving the surrounding world, processing the available information, and interacting with people (Grzadzinski et al. 2021). In toddlers, ASD is often characterized by social communication challenges (Bradshaw et al. 2021), such as an absence of facial connection, speech milestones not being reached by a certain age, understanding simple gestures, not showing an interest in active play, and a decreased interest in talking (Craig et al. 2021). These warning signs are crucial during the early years of life, which is the turning point of cognitive, emotional, and language skills development (Reetzke et al. 2022). In the absence of timely intervention, ASD can pose a significant barrier for a child in attaining age‐appropriate social, language, and environmental adaptation skills, which can lead to chronic academic and social challenges in the future. In any case, as research suggests, early and sustained behavioral and educational therapies significantly improve developmental milestones (Weitzman et al. 2022). Likewise, there is a risk that a toddler with ASD will miss the intervention, and as a consequence, there is the risk of underdevelopment that will require lifelong specialized support. In this case, the scientific and social challenges improve diagnosis for early‐aged children by working on accuracy. ASD diagnostic tools pose not only a scientific challenge, but a societal challenge as well. Immediate consequences on the long‐term well‐being of children and their families' quality of life are not to be understated (Riera‐Negre et al. 2024).

Therefore, it is essential to combine precision and speed when addressing the concerns of ASD detection in toddlers (Nawghare and Prasad 2025). Relying too much on subjective assessment is not an option. Employing advanced machine learning (ML) and optimization methodologies can solve these problems (Rahman et al. 2025). Comprehensive analysis of behavioral questionnaires, speech, and even neuroimaging can and will yield critical information disguised in complexity and subtlety. Such systems can provide information that is not readily available in clinical assessments. Families will be able to obtain a diagnosis of ASD much faster and, as a result, begin targeted treatment much earlier (Qin et al. 2024). The goal of this early, proactive intervention is to fast‐track speech, social, and appropriate learning skill development, which in turn supports the child's emotional development and overall learning capability as they grow. Such tools provide automation, and AI‐driven systems can be implemented even when resources and personnel are limited (Younes et al. 2025). Therefore, the availability of a reliable ASD diagnosis expands. Powerful tools have the potential to significantly change the ASD child's development path and reduce the need for future intervention, increasing autonomy and life quality (Musetti et al. 2021).

The relevance of ASD in toddlers goes beyond its potential developmental consequences; it underscores the necessity for ASD detection to be both timely and precise (Kohli et al. 2022). Access to critical interventions during the most crucial years of brain development can profoundly change life outcomes. Although traditional diagnostic methods have their merits, reliance on specialized diagnostic knowledge and lengthy evaluation periods hampers their efficiency and accessibility (Rubio‐Martín et al. 2024). There is a pressing need for highly sophisticated, intelligent, and data‐driven solutions that enable precise screening and rapid, wide‐scale evaluation. The need for early ASD detection inspires the current study, which aims to integrate advanced computational models with early detection technology (Jarrah and Abu‐Khadrah 2024). Bridging these domains provides the ASD detection technology with the strength and accessibility that enhance early intervention outcomes and mold toddlers' developmental trajectories toward a healthier path in life. To overcome these obstacles, this study proposes a glowworm swarm optimization–extreme learning machine (GSO‐ELM) framework, which integrates feature selection using swarm intelligence with a fast, generalized single hidden layer ELM. GSO successfully searches through the feature space to identify the most discriminative attributes while also optimizing the ELM's hyperparameters, which balances the model's complexity and accuracy. The goal of this hybridization is to achieve the best possible classification results while also minimizing the model's training time and memory usage. The model is tested on the ASD benchmark dataset, and its results are compared with those of other cutting‐edge ML and deep learning (DL) models. The results showed that the GSO‐ELM model outperformed the others in terms of accuracy, AUC, and F1‐score while also significantly reducing the computational complexity, making it ideal for scalable and real‐time ASD screening systems.

## 2 Background Research Analysis

ASD is a neurodevelopmental disorder marked by difficulties in social engagement, interaction, and the presence of repetitive behaviors. Advances in automation, particularly through ML and DL technologies, are significantly enhancing ASD screening. Other studies have used neuroimaging, magnetic resonance imaging (MRI), functional MRI (fMRI), electroencephalogram (EEG), and even demographic information alongside behavioral and written questionnaires to construct ASD diagnostic models. This paper is a systematic review of studies focusing on ASD detection based on ML and DL technologies, outlining the relevant methodologies, contributions, and gaps of the examined studies. Mujeeb Rahman and Monica Subashini (2022) created a deep neural network (DNN) model for screening ASD using the QCHAT dataset. The model obtained a high classification accuracy by discerning feature patterns from the subjects' behavioral responses. The results underscore the potential DL algorithms have in the noninvasive early screening of ASD. The major limitation of the model is its reliance on a single behavioral questionnaire, which does not capture physiologic indicators.

Xu et al. (2021) applied a multilayer neural network to classify ASD via short‐term spontaneous hemodynamic fluctuations. Brain activity was captured using fNIRS, and pattern recognition was implemented using DL techniques. The model successfully differentiated ASD patients from control participants. One of the significant drawbacks is the need for specialized neuroimaging equipment, which significantly limits ASD screening in resource‐limited regions. Nogay and Adeli (2023) advanced the ASD diagnostic paradigm from structural brain MRI employing convolutional neural networks (CNNs) along with grid search optimization. The feature extraction performed by the CNN model on the MRI scans significantly improved the diagnostic accuracy. The investigation validated structural pattern identification with promising diagnostic accuracy. Still, MRI‐based methodologies are constrained by costs and limited availability for large‐scale screening.

Khudhur and Khudhur (2023) employed traditional ML algorithms such as SVM, Random Forest, and *k*‐NN, achieving varying accuracy levels for classifying ASD across increments of age. The ML methods did reveal age‐dependent differences in prediction performance. Still, the glaring inconsistency across age groups indicates refinement for specific developmental benchmarks. The enhanced convolution neural networks, designed by Kashef (2022) for the ASD diagnosis, attempt to foster both efficiency and precision. The model employed more sophisticated convolutional layers in an attempt to capture deeper input data patterns. Although the standard CNN's accuracy was surpassed, the model may still suffer from complexity‐driven overfitting and computational strain due to the lack of proper data. Al‐Qazzaz et al. (2024) applied transfer learning and hybrid deep CNNs for the classification of ASD using EEG signals. The application of transfer learning by fine‐tuning pretrained EEG models on EEG datasets improved accuracy and generalization. This method facilitated effective feature reuse and reduced training time. A primary challenge remains the extensive EEG signal preprocessing needed due to noise contamination. Abitha et al. (2022) implemented multi‐objective evolutionary optimization to refine an ANN for ASD classification. They used NSGA‐II for multi‐objective optimization, focusing on achieving a trade‐off between accuracy and complexity of the model. The model exhibited improved performance on the given class compared to the benchmarks. On the downside, evolutionary computation methods are notoriously inefficient and slow.

Sabegh et al. (2023) developed a new CNN architecture for detecting ASD using fMRI scans. The model performed well in automatically extracting relevant brain imaging spatial‐temporal indicators, showing strong diagnostic accuracy in differentiating ASD from neurotypical peers. Clinical practicality, however, is limited due to reliance on fMRI scans, which are costly and require substantial infrastructure. Uddin (2023) developed a feature‐optimized ML pipeline for predicting ASD in children and adults. Robust model feature selection improved the model's accuracy, and several classifiers were evaluated to enhance demographic adaptability. However, imbalanced datasets and cultural bias in data collection may limit the model's adaptability and generalization. Loganathan et al. (2023) created a bi‐gated recurrent unit (Bi‐GRU) model that uses chaotic optimization for ASD detection and classification. The model's stability was enhanced by an ensemble strategy that utilized weighted averages. The system was successful in capturing temporal and sequential data patterns. Deep ensemble models, however, remain challenging regarding their interpretability. Omotehinwa et al. (2024) performed hyperparameter tuning on a 1D CNN for early ASD diagnosis using Bayesian optimization. The model enhanced learning and precision by leveraging signal data. Accuracy for early detection was promising. In the diagnosis stage, the lack of a spatial dimension posed an interpretive hurdle for 1D CNNs relative to formats like MRI or fMRI.

Bhandage et al. (2023) applied an Adam war strategy optimized deep belief network to classify ASD. The author introduced a hybrid model that enhances convergence and precision for classification, and its performance was consistent and robust in a wide range of tests. Nonetheless, the applications of deep belief networks are limited by the need for training data and susceptibility to vanishing gradients in deep architectures. Almars et al. (2023) proposed the ASD2‐TL Gorilla Troops Optimizer (GTO) model, which combines transfer learning and GTO for ASD detection. This approach successfully leveraged pretrained models and utilized tuned parameters for optimized transfer learning, achieving high accuracy and robust performance. Regardless, the novelty of GTO demands broader investigation and validation across more diverse and larger datasets.

Wang et al. (2025) proposed a hybrid intelligent decision‐support framework that combines retrieval‐augmented generative models with structured knowledge graphs to improve decision accuracy, reasoning transparency, and context relevance across domains like healthcare and finance, demonstrating advantages over single‐approach systems but requiring complex orchestration mechanisms that could challenge deployment in resource‐constrained settings. Deng et al. (2025) investigated real‐time AI decision‐support systems leveraging low‐latency interpretable models and Edge–IoT integration to enable efficient, flexible human–AI cooperation, highlighting opportunities for scalable deployment while noting challenges in balancing model performance with interpretability in resource‐limited environments.

The above research work (Table 1), focusing on the detection of ASD, multimodally applies ML and DL techniques with an emphasis on accuracy, sensitivity, and specificity. Accuracy greater than 90% as reported in studies by Nogay and Adeli (2023) and Bhandage et al. (2023) confirms strong model performance. Evaluation techniques such as grid search, transfer learning, evolutionary multi‐objective optimization, and GTO have enhanced model convergence and predictive ability. Many approaches, however, lack flexibility and full clinical feasibility because of specialized equipment (e.g., fMRI or EEG), or are not age or dataset adaptable. Addressing this, Khudhur and Khudhur (2023) and Uddin (2023) focused on designing adaptable age models. From the various researchers' opinions, the exact problem involved in the ASD prediction is illustrated as follows.

**TABLE 1:** Analysis of existing researchers' works on ASD prediction.

Study	Accuracy	Sensitivity	Specificity	Reliability	Effect	Flexibility	Novel optimization	Clinical feasibility
(Mujeeb Rahman and Monica Subashini 2022)	92	↑	↑	↔	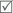	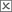	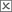	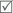
(Xu et al. 2021)	89	↔	↑	↑	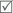	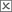	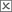	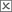 (Specialized equipment)
(Nogay and Adeli 2023)	94	↑	↑	↑	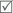	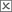	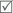 (Grid search)	↔
(Khudhur and Khudhur 2023)	88	↔	↔	↔	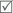	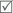 (Age groups)	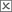	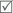
(Kashef 2022)	91	↑	↔	↑	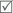	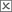	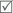 (Attention mechanisms)	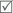
(Al‐Qazzaz et al. 2024)	93	↑	↑	↑	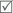	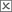	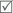 (Transfer learning)	↔
(Abitha et al. 2022)	90	↔	↑	↑	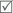	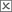	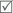 (Evolutionary MOO)	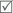
(Sabegh et al. 2023)	91	↑	↑	↑	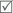	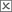	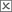	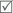 (fMRI constraints)
(Uddin 2023)	87	↔	↔	↔	↑	↑ (Age groups)	↑ (Feature optimization)	↑
(Loganathan et al. 2023)	93	↑	↑	↑	↑	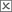	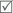 (Chaotic optimization)	↔
(Omotehinwa et al. 2024)	−(18% FP↓)	↑	↑	↑	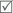	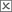	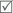 (Bayesian)	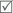
(Bhandage et al. 2023)	94	↑	↑	↑	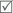	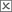	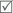 (Adam war)	↔
(Almars et al. 2023)	90	↑	↔	↑	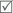	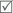 (Cross‐dataset)	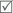 (GTO)	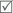

*Note*: 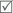:yes, 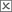: no, ↑: high, and ↔: moderate.

### 2.1 Research Challenge

The traditional ASD faces the main challenges in accurately distinguishing between the toddler's development and behavioral patterns because of the limited and imbalanced screening information. The ASD detection systems face task complexity due to low interclass overlap (between non‐ASD and ASD traits) and high intraclass variability (deviation among ASD individuals), which creates complexity during task classification. Consider $X=\{x_{1},x_{2},\text{…},x_{n}\}$ is the toddler input features obtained from the student behavioral features like age, ethnicity, and A1–A10 response; $Y=\{y_{1},y_{2},\text{…},y_{n}\}$ is the ASD classification labels $y_{i}\in \left\{0,1\right\}$ where 0 means no and 1 means yes. The system intends to reduce the misclassification rate by utilizing the f:X→Y function that predicts the ASD outputs, such as ${\hat{y}}_{i}=f\left(x_{i}\right)$. During the mapping process, the model must address various obstacles, including limited data and class imbalance issues. Initially, the dataset has a class imbalance problem in which P(y=1)”P(y=0) that causes biased learning. In addition, the dataset consists of noisy information, missing details, and subjective questionnaires related to the response that cause the inconsistent values in X. Therefore, the learned hypothesis (f) may be a cause of the overfit issue on unknown data. Finally, the standard recognition model faces difficulties in managing the high true negative rate and true positive rate, which requires an effective and optimized model $\Theta \subseteq R^{d}$ for updating the parameters θ∈Θ to convert the model into a computationally intensive one with overlapping and labeled data. Thus, the primary research issue is constructing a clear, strong, and effective learning model $f_{\theta }$ improving a mixed objective criterion, which includes accuracy, general position, and general learning efficiency, and at the same time handling data sparsity, noise, and real‐world variability. This requires a new learning paradigm with built‐in optimization that adaptively balances parameter search with the need for ASD‐positive samples to achieve reliable and unbiased prediction.

### 2.2 Research Objectives

The primary research objectives are framed as follows:

- To improve autism spectrum prediction accuracy and ensure generalization by analyzing the unknown data to minimize overfitting issues in high‐dimensional feature spaces.
- To optimize the ASD model sensitivity–specificity trade‐off by fine‐tuning the network parameters to maximize the optimistic case prediction without losing the false negative control.
- To reduce the data imbalance issues via the ELM loss $\min_{\theta \in \Theta }\sum_{i=1}^{n}w_{y_{i}}\cdot L\left(f_{\theta }\left(x_{i}\right),y_{i}\right)$ to reduce the bias in predictions.
- To balance prediction accuracy, computational efficiency, and sensitivity, ensuring fast predictions and robustness for clinical use.

The rest of the paper is then organized as follows:

Section 1 presents a basic discussion on ASDs in toddlers. Section 2 describes relevant studies. Section 3 explores the working process of the Glowworm Optimization with Extreme Learning Machine Networks (GO‐ELMN) model, and the efficiency of the GO‐ELMN is analyzed in Section 4. Section 5 presents the conclusion.

## 3 ASD Prediction

**FIGURE 1:**
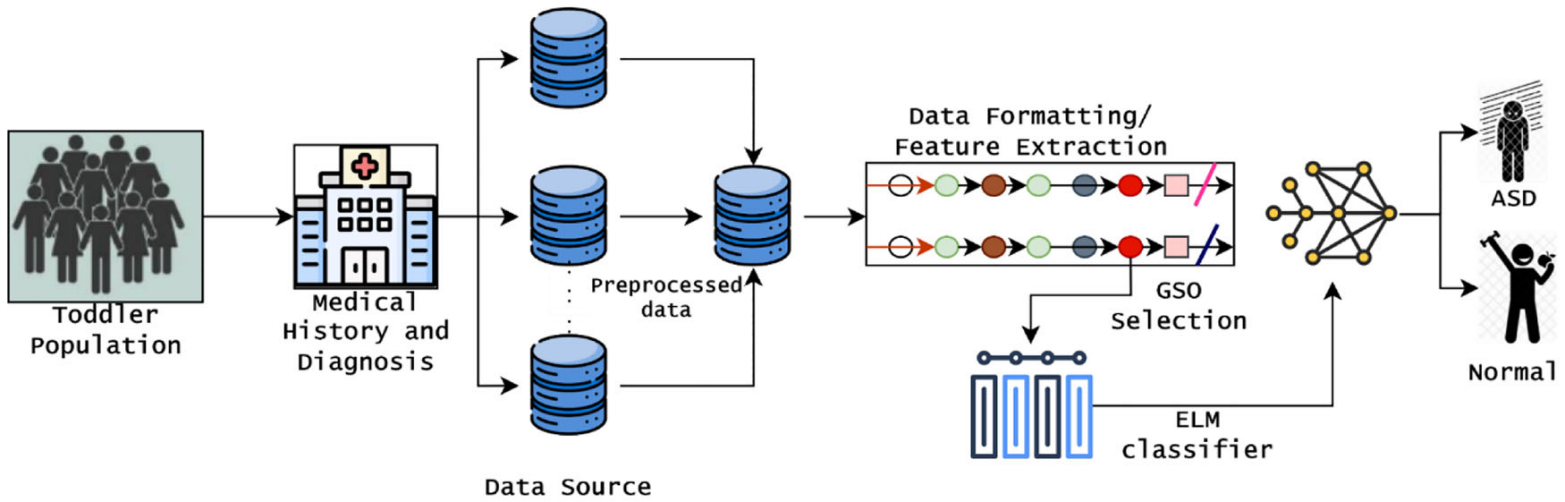
Architecture for ASD prediction.

This work aims to develop an accurate, scalable, and intelligent predictive model for identifying ASD in children through the application of effective screening techniques. The conventional prediction techniques depend on subjective, time‐intensive, and difficult‐to‐access patient data in a restricted resource environment. The challenges are addressed by introducing the optimized ELMN, which are capable of processing limited data and effectively solving class imbalance issues. During the prediction, the model instability is addressed by incorporating the glow swarm optimization technique that frequently updates the network parameters, which directly improves the classification accuracy and generalization. To improve the ASD prediction risk, the introduced approach explores children's behavioral, medical, and demographic features from the ASD screening dataset (Thabtah 2017). Then, the detailed working process and prediction procedure are illustrated in Figure 1, which provides fast and interpretable screening solutions to the pediatric healthcare environment with limited clinical expertise scenarios.

### 3.1 ASD Dataset

This study uses the ASD dataset (https://www.kaggle.com/datasets/fabdelja/autism‐screening‐for‐toddlers), which Dr. Fadi Fayez developed, and the information is gathered with the help of a mobile screening application. The dataset comprises 1054 instances and 19 features, encompassing both behavioral and demographic information. The primary objective of the data collection is to identify the risk factors associated with toddlers. The dataset consists of 10 binary variables gathered based on the Autism Toddlers Quantitative Checklist question. The questions are relevant to the toddler's communication, social interactions, attention behavior, pretend play, and pointing. The question receives responses like “never,” “rarely,” “usually,” or “always.” The demographic features, such as gender, age, ethnicity, birth jaundice history, country of residence, respondent relationship, and family ASD history, are gathered. Finally, the target binary label is included based on the questionnaire score value that is used to identify the ASD risk. Then, the sample dataset information is listed in Table 2.

**TABLE 2:** Sample ASD dataset information.

S. No.	Age (M)	QCHAT‐10‐score	Sex	Ethnicity	Jaundice	Family history with ASD	Who completed the test	Class/ASD traits
1	28	3	f	Middle Eastern	Yes	No	Family member	No
2	36	4	m	White European	Yes	No	Family member	Yes
3	36	4	m	Middle Eastern	Yes	No	Family member	Yes
4	24	10	m	Hispanic	No	No	Family member	Yes
5	20	9	f	White European	No	Yes	Family member	Yes
6	21	8	m	Black	No	No	Family member	Yes
7	33	5	m	Asian	Yes	No	Family member	Yes
8	33	6	m	Asian	Yes	No	Family member	Yes
9	36	2	m	Asian	No	No	Family member	No
10	22	8	m	South Asian	No	No	Healthcare professional	Yes
11	36	6	m	Hispanic	Yes	Yes	Family member	Yes
12	17	8	m	Middle Eastern	Yes	No	Family member	Yes
13	25	0	f	Middle Eastern	Yes	No	Family member	No
14	15	7	f	Middle Eastern	Yes	No	Family member	Yes
15	18	0	m	Middle Eastern	No	No	Family member	No
16	12	7	m	Black	No	No	Family member	Yes
17	36	0	m	Middle Eastern	No	Yes	Family member	No
18	12	8	f	Middle Eastern	Yes	No	Family member	Yes
19	29	3	f	Middle Eastern	No	No	Family member	No
20	12	7	f	Black	No	No	Family member	Yes

Table 2 presents screen data for toddlers within this study, including a subset of behavioral, demographic, and clinically relevant autistic features for toddlers within a given age range of 16–30 months. This dataset is structured to align with the proposed GO‐ELMN prediction framework. Each entry in the dataset corresponds to a toddler and is described by quantitative and categorical features like age in months, QCHAT‐10 score (an autism screening test score ranging from 0 to 10), biological sex, ethnicity, history of neonatal jaundice, family history of ASD, and the test administrator's relationship to the child. The screening result is represented by a binary target variable (class/ASD traits), with “Yes” indicating potential ASD traits and “No” indicating no detected ASD tendencies. From a dataset construction standpoint, the numerical features (age and QCHAT‐10 score) are normalized, and categorical features are one‐hot encoded. The GO‐ELMN pipeline starts with feature extraction and refining feature importance, subsequently mapping input vectors $x\in R^{n}$ to a fully optimized ELM hidden layer topology. Glowworm optimization concurrently optimizes the network hyperparameters (hidden neuron size [h], activation parameters [α]) while targeting classification accuracy (A), minimizing loss value (L), and enhancing the receiver operating characteristics for balancing the specificity and sensitivity. The process guarantees the system understands complicated mappings relating clinical or demographic details to ASD outcomes. It also leads to the development of a quickly converging and high‐accuracy screening tool that can be used in pediatric healthcare settings.

### 3.2 Data Processing and Formatting

The data refinement phase lays out the groundwork of the GO‐ELMN‐based ASD prediction system, guaranteeing that raw screening data are transformed into a high‐fidelity dataset that is free from extraneous noise, is normalized, and can be understood by machines before applying the model training. Within real‐world clinical datasets such as toddlers' screening records for autism, data are not only sparse but also rife with out‐of‐order fields, inconsistent data entry styles, and irrelevant data. If such data are provided to ML models, the system will be overly perfectionist, leading to excessively biased learning and issues with generalization and prediction accuracy. The refinement phase makes use of statistically informed data imputation, feature transformation, normalization, and resampling to solve the previously mentioned issues. In this phase, extrinsic noise is minimized, dimensionality is reduced, and intrinsic patterns within the data are preserved. For the given dataset, $D={\left\{\left(x_{i},y_{i}\right)\right\}}_{i=1}^{N}$; $x_{i}$ consists of both categorical ($c_{i}$) and numerical ($n_{i}$) attributes. The attributes in D are fed into the preprocessing process and applied to the sequence of transform function $T:R^{n}\cup C^{m}\to R^{p}$ to get the output feature set 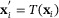, which is normalized and complete to predict the ASD with maximum accuracy. The $n_{i}$ consists of the QCHAT‐10 score and age‐related attributes, and the $C_{i}$ attributes consist of family history, ethnicity, and sex related details that include missing values, which leads to statistical bias and minimizes the learning process efficiency. Therefore, the missing values ($m_{i}$) are addressed by applying systematic imputations that replace inconsistent or missing values, thereby managing the distributed data. For given, $n_{i}$ attributes j the $m_{i}$ is computed, which is replaced using the mean value that is represented as $x_{ij}\leftarrow \frac{\sum_{k=1}^{N}x_{kj}\cdot \delta_{kj}}{\sum_{k=1}^{N}\delta_{kj}}$; here, for the sample, the *i*th feature's *j* value is represented as $x_{ij}$. During this computation, $\left\{ \begin{array}{c} \mathrm{if}\;x_{\mathrm{kj}}\neq \mathrm{NAN}\;\;\delta_{\mathrm{kj}}=1\\ \mathrm{othe}\mathrm{rwise}\;\;\delta_{\mathrm{kj}}=0 \end{array}\right.$ is considered to eliminate the $m_{i}$ value that ensures the central tendency of the data and reduces the distribution distortion. For the $C_{i}$ value, the missing values are computed, which is changed to the most frequent category that is defined as $x_{ij}\leftarrow \mathrm{mode}\left(x_{\cdot j}\right)$. Here, the $\mathrm{mode}(x_{\cdot j})$ gives the category value that has the highest frequency in j, which manages the $C_{i}$ value‐related modal distribution that helps to avoid the class imbalance issues effectively. Then the combination of missing values is integrated, which is defined as $T_{\mathrm{impute}}:R^{n}\cup C^{m}\to R^{n}\cup C^{m},$ and the $T_{\mathrm{impute}}$ changes the NAN numerical features with mean imputation and categorical features with mode imputation. The $T_{\mathrm{impute}}$ imputation process manages the fully dense feature space, which ensures the ASD prediction reliability and smooth optimization. After handling $m_{i}$, the $c_{i}$ such as sex, ASD family history, jaundice, ethnicity, sex, and test completed features are changed into a numerical representation. In the search space $R^{d}$, the categorical feature $F_{\mathrm{cat}}=\left\{f_{1},f_{2},\text{…},f_{C}\right\}$ is processed, and the finite set of values $V_{j}=\left\{v_{1},v_{2},\text{…},v_{K_{j}}\right\}$ is obtained. First, the nominal $c_{i}$ such as ethnicity and sex features are processed using a one‐hot encoding process that transforms the category into a binary vector, which is represented as $\phi_{\mathrm{onehot}}\left(v_{k}\right)={\left[0,\text{…},0,1,0,\text{…},0\right]}^{⊤}\in {\left\{0,1\right\}}^{K_{j}}$. Consider the example, Sex∈{Male,Female} then the encoding is defined as Male→[1,0],Female→[0,1] and the final transformation of $c_{i}$ is defined as $x_{ij}\mapsto e_{K_{j}}\left(k\right),\mathrm{where}\;k\;\mathrm{is}\;\mathrm{the}\;\mathrm{index}\;\mathrm{of}\;\mathrm{the}\;\mathrm{category}\;x_{ij}$ and the $e_{K_{j}}\left(k\right)$ is represented as kth canonical bias vector of $R^{K_{j}}$. Then, the Boolean attributes, such as ASD family history and jaundice, are mapped to the encoding defined as Yes→1, No→0. The binary encoding is represented as $x_{ij}\to I$ *x_ij_* = “Yes”; here, indicator function is represented as I(·). This process is performed on the entire dataset *D*, which has *m* attributes, and the new dimensionality of the features is represented as $M^{\prime }=M_{\mathrm{num}}+\sum_{j=1}^{C}K_{j}$; here, the number of numerical features is represented as $M_{\mathrm{num}}$ and the distinct categories features are represented as $K_{j}$ and the transformation is defined as $T_{\mathrm{encode}}:X\to R^{M^{\prime }}$. Then, data normalization is applied, changing the heterogeneous features into stable numerical values, which are highly utilized in extreme learning networks. For every feature $\left\{x_{1j},\text{…},x_{Nj}\right\}$, the scaling statistics are computed on the training set by applying the deterministic map ($S_{j}\left(\cdot \right)$) which converts the features into the respective numerical properties (${\tilde{x}}_{ij}=S_{j}\;\left(x_{ij}\right))$. The scaling process is performed by estimating the minimum ($\min_{k}x_{kj}$) and maximum value ($\max_{k}x_{kj}$) which is defined as ${\tilde{x}}_{ij}=\frac{x_{ij}-\min_{k}x_{kj}}{\max_{k}x_{kj}-\min_{k}x_{kj}}$. Then, the standard scaling (Equation 1), robust scaling (Equation 2), and unit‐norm scaling are applied to format the inputs.

(1) $$\left.\begin{array}{c} \begin{array}{c} \;\mu_{j}=\frac{1}{N}\;\sum_{k=1}^{N}x_{kj},\\ \;\sigma_{j}=\sqrt{\frac{1}{N}\sum_{k=1}^{N}{\left(x_{kj}-\mu_{j}\right)}^{2}}\; \end{array}\\ \;{\tilde{x}}_{ij}=\frac{x_{ij}-\mu_{j}}{\sigma_{j}}\;. \end{array}\right\}$$

(2) $$\left.\begin{array}{c} \;\mathrm{me}d_{j}=\mathrm{median}\left(x_{\cdot j}\right)\\ \;\mathrm{IQ}R_{j}=Q3_{j}-Q1_{j}\\ \;{\tilde{x}}_{ij}=\frac{x_{ij}-\mathrm{me}d_{j}}{\mathrm{IQ}R_{j}}\;, \end{array}\right\}$$

Equation (2) is used when the features have different variances, and Equation (2) preserves the central tendency. Along with this, unit‐norm scaling is computed as ${\tilde{x}}_{i}=\frac{x_{i}}{∥x_{i}{∥}_{2}}\;,$ which is helpful while the classifier must be invariant to global vector magnitude. The normalized inputs $\left(X_{\mathrm{norm}}\right)$ guarantee that the hidden output matrix H with entries $H_{ij}$ possesses columns with well‐conditioned geometry. Well‐conditionedness ([𝐻] small) is beneficial for the stability of computations involving the Moore–Penrose inverse *H* ^†^ and for the stability of heuristically and optimally calibrated closed‐form solutions for the output weights. When applying SMOTE resampling, first perform a train/test split, then apply normalization to the training set. The scaler fitted on the training set can then be used to transform the synthetic samples and the test set. Alternatively, normalization can be done first, then SMOTE, but the scaler must only be fitted to the original training data to prevent leakage. For categorical one‐hot encoded columns, the binary values of {0,1} can either be left as is or added to the scaling pipeline without being affected, since min–max scaling preserves these values. Also, do not apply a *z*‐score to sparse one‐hot vectors. Lastly, the choice of normalization should match the activation function: for min–max or scaled to [−1, 1] for sigmoid and tanh, *z*‐score or linear ELMs. This decision should be treated as a hyperparameter and optimized using GSO for best performance in the upstream tasks. According to the discussions, the pseudocode involved in this preprocessing step is shown in Algorithm 1.

ALGORITHM 1 **Preprocessing.**

**Table:** 

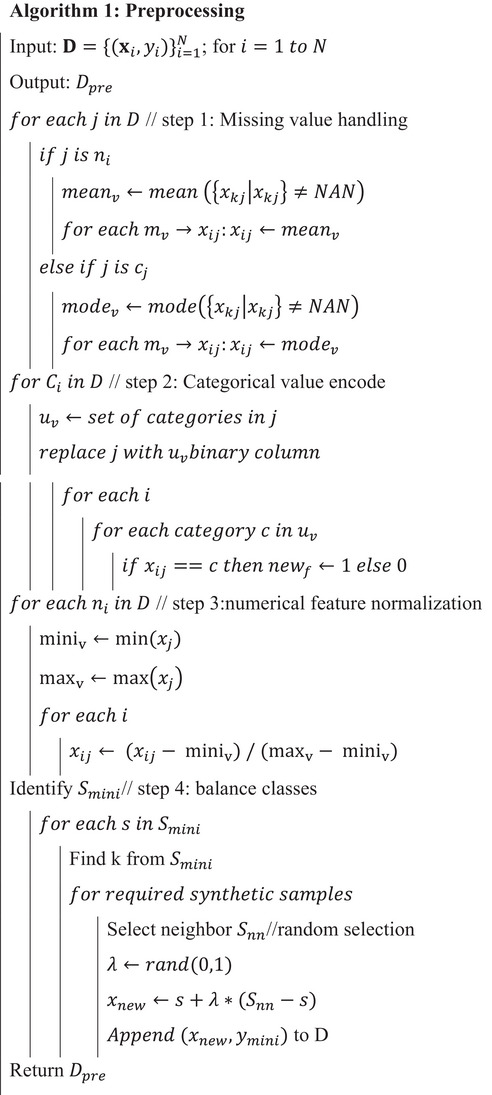

As a result of the preprocessing pipeline, the dataset is now a complete and structured numerical dataset, containing samples represented as normalized feature vectors with binary‐encoded categorical variables. This process creates a balanced measurement of features on a ratio scale, preserves ordinality and categorical bias, and does not encode missing values. Normalized feature scaling and preserving categorical semantics ensure ordinality bias is maintained. With the dataset now transformed into a consistent numerical format, it is prepared for ingestion into the GO‐ELMN model. At this point, each record can be understood as an n‐dimensional input vector, which can be optimized and learned in the subsequent phases of feature selection and classification learning.

### 3.3 Autism Attribute Selection

Following preprocessing, every sample appears as a numerical vector $x_{i}\in R^{d}$ is processed by a feature selection approach to select the optimal features. In the feature‐selection phase, the aim is to determine a minimal subset among the d features that maximize the classifier's accuracy on ASD detection while avoiding excessive redundancy and overfitting. In the GO‐ELMN pipeline, this study features a selection performed by applying the GSO algorithm to solve optimization problems. In this case, the fitness of a candidate subset is determined by training an ELM on the subset and evaluating its performance during cross‐validation. The preprocessed data $D_{\mathrm{pre}}$ is the input to this phase, which is formalized as $D={\left\{\left(x_{i},y_{i}\right)\right\}}_{i=1}^{N}$; here $x_{i}\in R^{d}\;and\;y_{i}\in \left\{0,1\right\}$. The gathered $x_{i}\in R^{d}$ is processed over the binary mask $m\in {\left\{0,1\right\}}^{d}$ to minimize the feature dimensionality $x_{i}^{\left(m\right)}=x_{i}\;⊙m$ with the size of $∥m\;{∥}_{1}=\sum_{j=1}^{d}m_{j}$. Then the primary intention of this phase is represented in Equation (3).

(3) $$\left.\begin{array}{c} m^{*}=\arg\;\underset{m\in {\left\{0,1\right\}}^{d}}{\max\;J(m)}\\ J\left(m\right)=\mathrm{Scor}e_{\mathrm{CV}}\left(m\right)-\alpha \frac{{\left\|m\right\|}_{1}}{d}. \end{array}\right\}$$

**FIGURE 2:**
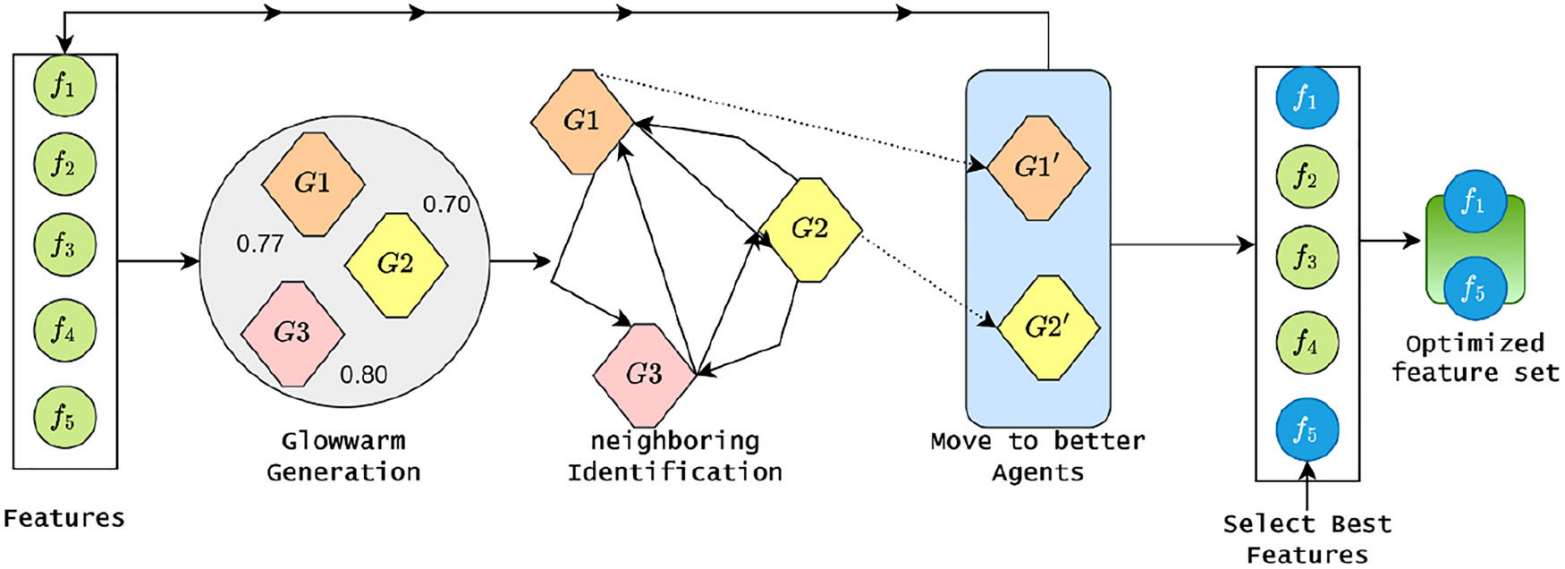
Process of GSO‐based feature selection.

In  Equation (3), the $\mathrm{Scor}e_{\mathrm{CV}}\left(m\right)$ value is obtained during the extreme learning training on $x^{\left(m\right)},$ which is used to obtain the system's robustness. The subtraction term serves to penalize model complexity, thus leading to better optimization of smaller, more interpretable feature sets. As a matter of fact, $\mathrm{Scor}e_{\mathrm{CV}}$ is estimated for k−fold validation to eliminate the biased selection. Inner folds assess candidate masks, and outer folds estimate unbiased generalization. During this process, the scalar weight (α∈[0,1]) trade‐off between raw predictive power and model parsimony, a more feature‐dense model. As the search area is combinatorial (masks), either gradient‐free global optimizers (e.g., GSO) or continuous relaxations are used. Each $s\in R^{d}$ is transformed m via a transfer function (sigmoid plus threshold or probabilistic sampling) and fitness evaluations employing J(m). Implementation notes are as follows: (1) if the class distribution is skewed, employ imbalance‐aware $\mathrm{Scor}e_{\mathrm{CV}}$ (AUC or balanced accuracy); (2) to avoid semantically invalid partial encodings, preserve one‐hot group loss by selecting at the group level; (3) reduce computation through parallel population evaluations; (4) quantify selection stability by repeating the search with different seeds and report feature selection frequencies of $\left(\mathrm{Scor}e_{\mathrm{CV}},∥m{∥}_{1}\right)$. This formulation offers a tunable trade‐off of accuracy and simplicity, respecting ELM pretraining and the clinical interpretability of the model. Then the process of feature selection is illustrated in Figure 2.

According to Figure 2, the features are selected based on the fitness function that is computed from the ELM training and the binary mask (m). As mentioned, the selection process reduces the overfitting issue and ensures cross‐validation efficiency. For m, the sample dimensionality is reduced by element‐wise multiplication $x_{i}\left(m\right)=x_{i}\;⊙m$. During this process, the learning machine utilizes the L neurons with sigmoid activation (g(·)) function to estimate the fitness value. The hidden layer ($H\in R^{N\times L}$) computation is represented as $H_{i,j}=g\left(w_{j}^{⊤}x_{i}\left(m\right)+b_{j}\right),i=1,\text{…},N,j=1,\text{…},L$; here, the $w_{j}\in R^{d}$ is defined as a randomly defined input weight value, $b_{j}$ is a randomly defined bias value, and the activation function is represented as $g\;\left(z\right)=\frac{1}{1+e^{-z}}\;.$ The learning network uses the two conditions while defining the fitness values, which are defined as follows.

- *Random initialization condition*: $w_{j}\;\mathrm{and}\;b_{j}$ values are static after defining the parameters, which helps to determine the hidden layer with m.
- *Sparsity*: when $x_{i}\left(m\right)$ is the sparse while m excludes too many features $|m{|}_{1}”d,$ which accelerates the computations.

By considering these conditions, the output weight $B\in R^{L\times C}$ is estimated to understand classes learned via the regularized least squares value, which reduces the error value. Then the output is computed as $B={\left(H^{⊤}H+\lambda I\right)}^{-1}\;H^{⊤}T$; here, $T\in R^{N\times C}$ is defined as the one‐hot encoded target labels, ridge regularization parameters are defined as λ≥0, and the identity matrix is represented as I. Afterward, the $\mathrm{Scor}e_{CV}\left(m\right)$ value is evaluated using the *k*‐fold cross‐validation, in which the dataset is divided into *k*‐folds to preserve the class distribution. During this analysis, the $H_{i,j}$ computation consists of $O(N\cdot L\cdot |m{|}_{1})$ complexity, ridge regression has $O(L^{3}+L^{2}N)$ per fold, and the cross‐validation has O(k·ELMcost) computation complexity. From the analysis, the $\mathrm{Scor}e_{CV}\left(m\right)=\frac{1}{k}\;\sum_{i=1}^{k}\mathrm{Metric}\left(D_{k}\right)$ is considered the fitness value that helps to identify the optimized features from the dataset. After selecting the fitness value, the GSO approach is applied for choosing the binary features by mapping the agent position to the feature mask. The feature selection is performed according to the luciferin attraction and neighborhood cooperation.

#### 3.3.1 Continuous to Binary Mapping

The optimization algorithm works effectively in the continuous space, for every glowworm (i) has a position vector ($s_{i}\in R^{d}$); here, *d* is the number of features in the search space. The position vector ($s_{i}$) is to binary mask ($m_{i}\in {\left\{0,1\right\}}^{d}$) for making the effective feature selection. During this process, sigmoid thresholding is applied to get the mask value ($m_{i}$) which is defined in Equation (4).

(4) $$\left.\begin{array}{c} \;m_{i,j}=\left\{\begin{array}{cc} 1 & \mathrm{if}\;\sigma \left(s_{i,j}\right)>\tau ,\\ 0 & \mathrm{otherwise}, \end{array}\right.\;\\ \;\sigma \left(z\right)=\frac{1}{1+e^{-z}}\;. \end{array}\right\}$$

In Equation (4), the threshold value is τ∈(0,1)indefault0.5 and the $s_{i,j}$ is defined as the propensity used to choose the features (j).

#### 3.3.2 Agent State and Dynamics

After converting to $m_{i,j}$, each i state and dynamics are evaluated at iteration (t), which is characterized by position ($s_{i}\left(t\right)$), luciferin ($l_{i}\left(t\right)$), and neighborhood radius ($r_{i}\left(t\right)$). In these characteristics, $l_{i}\left(t\right)$ is defined as the fitness attractiveness, which combines the current performance and fitness decay, which is represented as $l_{i}\;\left(t+1\right)=\left(1-\rho \right)\;l_{i}\left(t\right)+\gamma J\left(m_{i}\left(t\right)\right).$ Here, ρ∈(0,1) is represented as a decay rate that has a 0.4 value, γ>0 is a gain factor that is used for fitness scaling ($J(m_{i}\left(t\right))$). The main intention of this phase is to identify the glowworms based on their luciferin value $J(m_{i}\left(t\right))$).; A glowworm with a higher $J(m_{i}\left(t\right))$ emits a stronger signal, thereby influencing the behavior of other glowworms. According to the signal, neighborhood worms are searched in the radius ($r_{i}\left(t\right)$) that is done as $N_{i}\left(t\right)=\left\{j\neq i|∥s_{j}\left(t\right)-s_{i}\left(t\right)∥\leq r_{i}\left(t\right)\;\mathrm{and}\;l_{j}\left(t\right)>l_{i}\left(t\right)\right\}.$ After selecting the neighboring worms, the movement to the neighbor $j\in N_{i}\left(t\right)$ probability is estimated using Equation (5).

(5) $$\left.\begin{array}{c} p_{ij}\;\left(t\right)=\frac{l_{j}\left(t\right)-l_{i}\left(t\right)}{\sum_{k\in N_{i}\left(t\right)}\left(l_{k}\left(t\right)-l_{i}\left(t\right)\right)}\;.\\ s_{i}\left(t+1\right)=s_{i}\;\left(t\right)+\beta \frac{s_{j}\left(t\right)-s_{i}\left(t\right)}{∥s_{j}\left(t\right)-s_{i}\left(t\right)∥}+\varepsilon \left(t\right) \end{array}\right\}$$

According to the probability in Equation (5), the higher $l_{j}$ is followed, and the movement is updated when j is selected, then i moves to the j, which is defined as $s_{i}\left(t+1\right)$; here, β>0 and the small random noise is represented as ε(t) which leads to exploration. Finally, radius adaptation ($r_{i}\left(t\right))$ is performed that dynamically adjusts to manage the $n_{t}$ for getting the neighbors, which is computed from the maximum radius ($r_{s}$), adaptation rate (α). Then the $r_{i}\left(t\right)$ is estimated from Equation (6).

(6) $$\left.\begin{array}{c} r_{i}\left(t+1\right)=\min\left(r_{s},\max\left(0,r_{i}\left(t\right)+\alpha \left(n_{t}-|N_{i}\left(t\right)|\right)\right)\right),\\ \mathrm{if}\;|N_{i}\left(t\right)|<n_{t}\;\mathrm{increases}\;r_{i}\\ \mathrm{else}\;\mathrm{shrink}\;\mathrm{it} \end{array}\right\}$$

#### 3.3.3 Group‐Wise Selection

With respect to one‐hot encoded features such as ethnicity and sex, picking out single columns is conceptually incorrect. Instead, a group mask $u\in {\left\{0,1\right\}}^{G}$ as a binary vector for *G* feature groups. This is further transformed into m=expand(u), which enforces group‐level selections as opposed to individual column selections. The search space for GSO operates in $R^{G}$ in contrast to $R^{d}$, which significantly reduces the number of dimensions. As an example, consider the “Ethnicity” feature, which is one‐hot encoded into five columns. By setting $u_{\mathrm{ethnicity}}=1$, all five columns are enabled, which maintains logical consistency in feature selection. This process is repeated until the fitness value $J\left(m\right)=\mathrm{AU}C_{\mathrm{CV}}\;\left(m\right)-\alpha \frac{|m{|}_{1}}{d}$ and it gives effective solutions for various complexity levels. Based on the above discussions, the pseudocode for the feature selection process is shown in Algorithm 2.

ALGORITHM 2 **GSO‐feature selection.**

**Table:** 

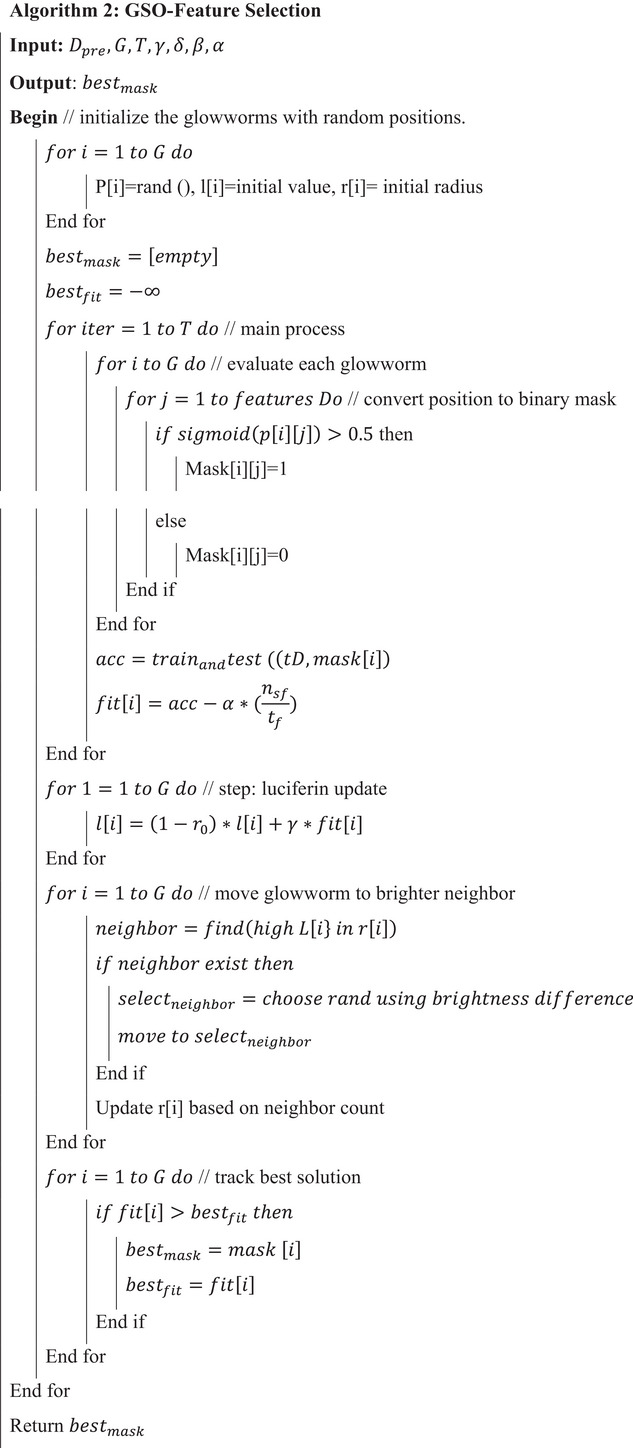

In a typical practical setting, the GSO parameters are set to balance exploration and convergence speed. For mid‐dimensional feature spaces, a population size G of 20–50 agents is adequate, with iterations 𝑇 set to a range of 30–100 and terminating if the best cross‐validated score stagnates. Luciferin decay is usually selected from the range of 0.3–0.5, while luciferin gain is set to 0.4–0.8 so that the fitness signal is stable and responsive. The agent movement step size (𝛽) is bounded from 0.03 to 0.2, with lower values preferred for high‐dimensional (𝑑) spaces to reduce unreliable jumps. The initialization radius for the neighborhood $r_{0}$ is set relative to the normalized search space, for example, $0.1\times \sqrt{d}$, with neighborhood radius scaling $r_{s}$ between 0.3 and 1.0. The desired neighbor count $n_{t}$ is set between 3 and 7 for meaningful local interactions. For the evaluator, ELM, the count of hidden neurons 𝐿 is set from 20 to 200, and the regularization coefficient is adjusted from $10^{-6}$ to $10^{2}$ exponentially. The optimization produces a final binary feature mask ($m^{★})$ in multi‐objective variants, a Pareto‐optimal set of masks, alongside their respective cross‐validated performance scores. To evaluate the selection stability, the counts of each feature over multiple runs are tallied. The ELM is then fully trained on the complete training set with $m^{★}$ and is tested on a separate holdout test set. After the evaluation, the features (or feature groups) are interpreted based on domain knowledge, particularly their clinical relevance in screening for ASD.

### 3.4 ELM‐Based ASD Prediction

**FIGURE 3:**
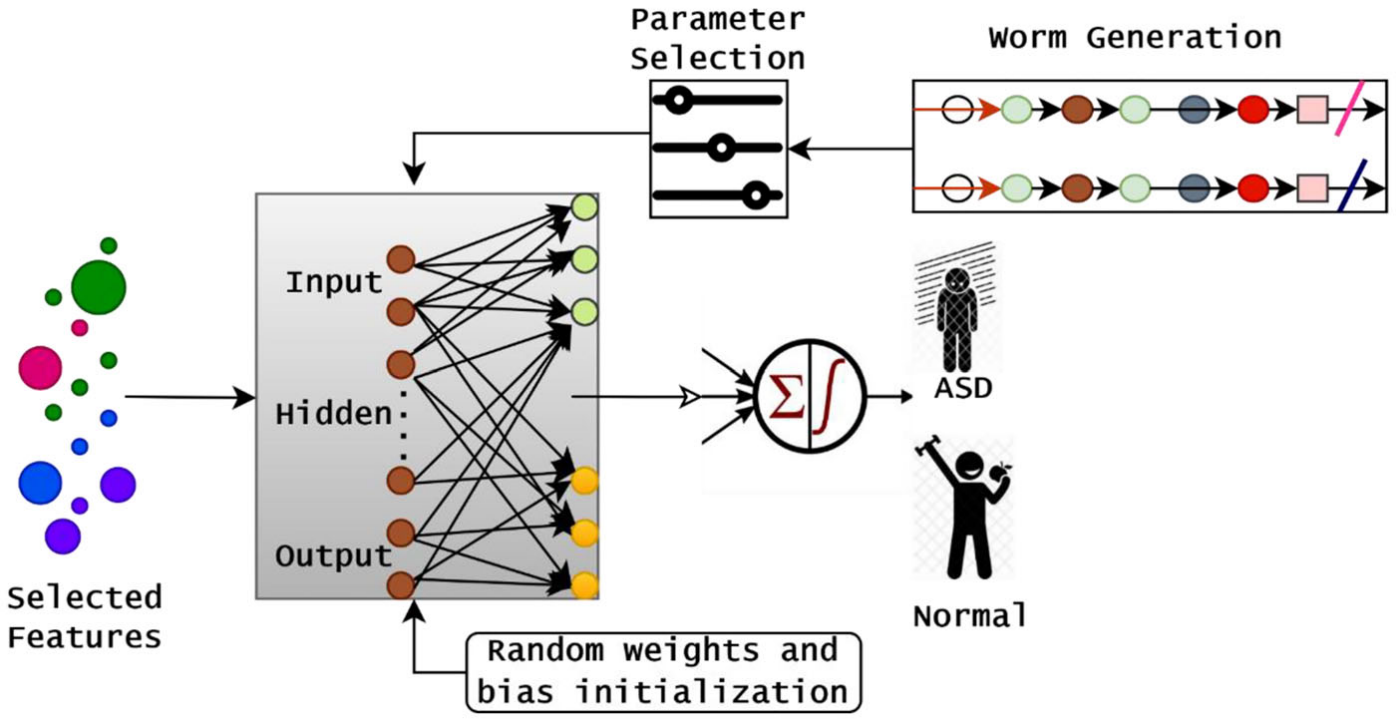
ELM network structure.

The previous step, such as preprocessing and feature selection procedure, gives normalized inputs $x_{i}\in R^{d}$ and binary mask ($m^{★}\in {\left\{0,1\right\}}^{d}$) which are processed further and reduce dimensionality ($d^{\prime }=∥m^{★}{∥}_{1}$) and minimized vector is defined as ${\tilde{x}}_{i}=x_{i}⊙m^{★}\in R^{d}.$ The vectors are fed into the ELMN to identify the toddler autism risk factor by mapping the $\hat{x}\mapsto \hat{y}$ with $\hat{y}\in \left\{0,1\right\},$ which improves the overall prediction accuracy on test data. During the analysis, the network uses a single hidden layer with L neurons and m outputs. Every layer has input weights that are defined as $W\in R^{L\times d^{\prime }}$, hidden biases ($b\in R^{L}$), and output weights ($B\in R^{L\times m}$) to get the optimized outputs. Then the structure of the ELM network is shown in Figure 3.

According to Figure 3, for all samples (*n*), the pre‐activation matrix is computed in the hidden units using Equation (7).

(7) $$\left.\begin{array}{c} H=g\left(Z\right)=g\left(XW^{⊤}+1_{N}b^{⊤}\right)\left(\mathrm{shape}\;N\times L\right)\\ Z=X\;W^{⊤}+1_{N}\;b^{⊤} \end{array}\right\}$$

In Equation (7), $XW^{⊤}$ gives the N×L matrix in which (i,j) entry is ${\hat{x}}_{i}^{⊤}w_{j}$; for all *N* rows, the broadcasted bias vector is represented as $1_{N}b^{⊤}$ and the column is $1_{N}\in R^{N}$. This computed elementwise activation is equivalent to the $H_{ij}=g\left(w_{j}^{⊤}{\hat{x}}_{i}+b_{j}\right)$. During this process, the radial basis function (RBF) uses the center value $C={\left[c_{1},\text{…},c_{L}\right]}^{⊤}\in R^{L\times d^{\prime }}$, which is computed along with the $\sigma_{j}>0$ width and the i,j element that is defined in Equation (8).

(8) $$\left.\begin{array}{c} \;H_{ij}=\exp\;\left(-\frac{∥{\hat{x}}_{i}-c_{j}{∥}_{2}^{2}}{2\sigma_{j}^{2}}\right)\;.\\ ∥{\hat{x}}_{i}-c_{j}\;{∥}_{2}^{2}=∥{\hat{x}}_{i}{∥}_{2}^{2}+∥c_{j}{∥}_{2}^{2}-2\;{\hat{x}}_{i}^{⊤}c_{j}. \end{array}\right\}$$

Here, the parameters are derived with the help of Equation (9).

(9) $$\left.\begin{array}{c} \;r_{i}=∥{\hat{x}}_{i}{∥}_{2}^{2}\;where\;r\in R^{N}\\ \;s_{j}=∥c_{j}{∥}_{2}^{2}\;where\;s\in R^{L}\\ K=XC^{⊤}\in R^{N\times L}\;with\;{\hat{x}}_{i}^{⊤}c_{j}\;entries \end{array}\right\}$$

Based on Equation (9), the square distance matrix (D) is estimated as $D=r1_{L}^{⊤}+1_{N}s^{⊤}-2K\;\mathrm{with}\;S\in R^{N\times L}$ and then it is defined as Equation (10):

(10) $$\left.H=\exp\;\left(-\;D⊘S\right)=\exp\left(-\frac{r1_{L}^{⊤}+1_{N}s^{⊤}-2XC^{⊤}}{\;2\;{\left[\sigma^{2}\right]}^{⊤}}\right)\right\}$$

In Equation (10), elementwise operation is referred to as ⊘ and 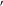 s vector is defined as $\left[\sigma^{2}\right]\in R^{L}$. The computed hidden *H* output is fed into the complete forward pass, and the network output is calculated as $\hat{T}=HB\left(\mathrm{shape}\;N\times m\right)$ and the probabilities for binary classification are defined as $P=\sigma (\hat{T})$ according to the ${\hat{y}}_{i}=1\left\{P_{i\cdot }\geq \tau \right\}$ decision rule. During this computation, the primary intention is to reduce the ridge objective that is derived via Equation (11).

(11) $$\left.\begin{array}{c} L\left(B\right)=∥HB-T{∥}_{F}^{2}+\lambda ∥B{∥}_{F}^{2}.\\ \left(H^{⊤}H+\lambda I_{L}\right)\;B=H^{⊤}T,\\ B={\left(H^{⊤}H+\lambda I_{L}\right)}^{-1}H^{⊤}T \end{array}\right\}$$

In Equation (11), instead of inverting an N×N matrix, smaller submatrices K are more efficient computationally. Matrix H captures all hidden activation outputs for the entire training dataset, making B computable by the closed form with non‐iterative, analytical, and instantaneous solutions characteristic of ELM. The computational burden for training is dominated by computing $O(NLd^{\prime })$ and solving the linear system. Then the regularization λ decreases variance as well as provides stability. It is advisable to apply the regularized form using faster linear‐algebra‐based solvers with no need to compute the inverse, and meter values explicitly. The inputs are normalized (already accomplished during preprocessing), the activation is computed, and L is determined by cross‐validation or a prior GSO‐based hyperparameter search. For prediction on new samples $X_{\mathrm{new}}\in R^{M\times d^{\prime }}$, we obtained $H_{\mathrm{new}}=g\left(X_{\mathrm{new}}W^{⊤}+1_{M}b^{⊤}\right)$. ${\hat{T}}_{\mathrm{new}}=H_{\mathrm{new}}\;B$ was predicted and then mapped to probabilities and a threshold for class labels. In the introduced ASD prediction framework, the function is responsible for shaping the hidden layer representation, which makes its choice particularly important. For sets of features with low dimensionality and a high proportion of categorical values, sigmoid activations are preferred. This is because those activations not only provide smooth nonlinear mappings, but are also bounded, which brings stability during training. Alternatively, if capturing local similarity structures is essential, for example, in cases where predictive feature clusters are involved, a RBF activation is helpful since it concentrates on nearness in the feature space. In a standard ELM model, the input weights 𝑊 and biases 𝑏 are usually randomly initialized, often from a uniform distribution in [−a,a]; in some implementations, biases may alternatively be set to small constant values. In this case, they are modified with GSO, which performs better by optimizing (a) the binary feature selection mask, (b) the hidden size 𝐿 and regularization 𝜆, or (c) parameters 𝑊 and 𝑏. This is to achieve a more robust hidden representation and reduce random sensitivity to initialization. The hidden size was set to and tuned in the range [20, 200], which expresses and balances the trained model with the computation cost and overfitting risk. Model regularization was introduced with the ridge parameter λ, which was searched logarithmically over $\left[10^{-6},10^{2}\right]$ to improve performance and generalization of the model. In addition to this, the pseudocode involved in the ASD prediction is shown in Algorithm 3.

ALGORITHM 3 **ELM‐ASD prediction pseudocode.**

**Table:** 

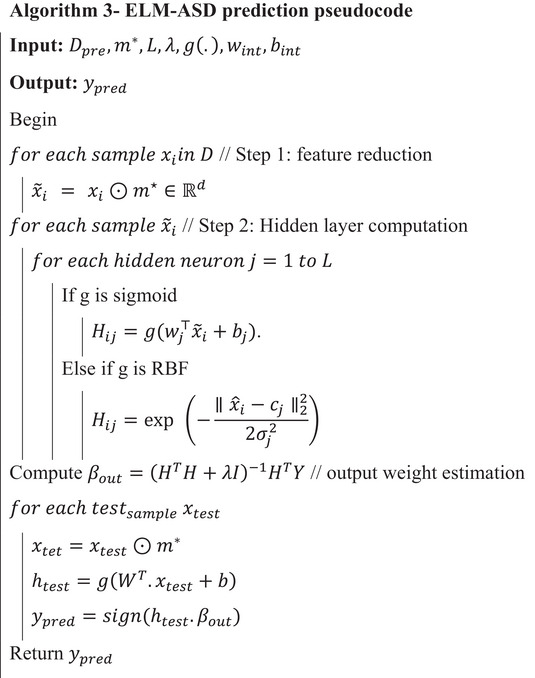

According to Algorithm 3, the output is predicted and then compared with the training set to identify any deviation between the output data and the training set. The training dataset comprises a collection of output labels for each sample, determining the error rate and prediction accuracy as the test data are explored. The deviation between the outputs is reduced with the help of an optimization technique. In standard ELM, Wandb are static after initialization, and β is computed from the statistical analysis. In contrast, the proposed framework integrates GSO‐based tuning for increasing prediction stability, which in turn improves parameter refinement. If Θ={W,b,L,λ} signifies the adjustable parameters, GSO modifies them iteratively as $\Theta^{\left(t+1\right)}=\Theta^{\left(t\right)}+\Delta \Theta^{\left(t\right)}$. Here, $\Delta \Theta^{\left(t\right)}$ is computed from the luciferin‐related neighboring search that is defined in Equation (12).

(12) $$\left.\begin{array}{c} \;l_{i}^{\left(t+1\right)}=\left(1-\rho \right)\;l_{i}^{\left(t\right)}+\gamma \cdot J_{i}^{\left(t\right)}\\ \Delta \;\Theta^{\left(t\right)}=\beta_{\mathrm{step}}\;\cdot \frac{\Theta_{\mathrm{neighbor}}^{\left(t\right)}-\Theta^{\left(t\right)}}{∥\Theta_{\mathrm{neighbor}}^{\left(t\right)}-\Theta^{\left(t\right)}∥} \end{array}\right\}$$

**FIGURE 4:**
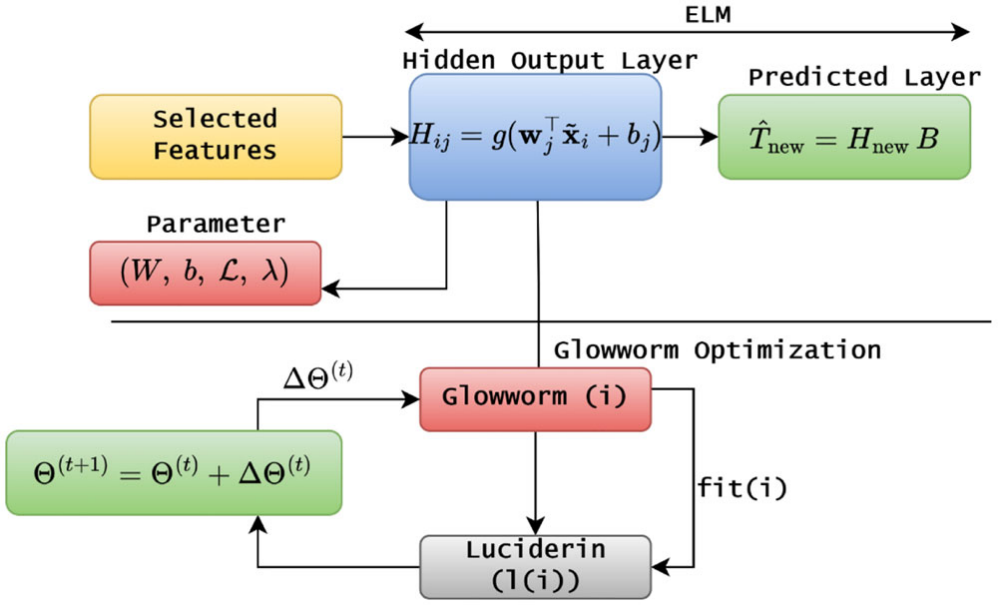
GSO parameter optimization in ELM.

In Equation (12) luciferin decay rate is defined as 𝜌, luciferin enhancement factor is represented as 𝛾, luciferin value is denoted as $l_{i}$, and fitness score $J_{i}^{\left(t\right)}$ parameters are related to chi‐squared fitness functions with luciferin values and fitness functions, 𝛽 step is the value controlling the movement step size in parameter space, which is the predicted step size of parameters. Overfitting is a problem of high model complexity and is countered with optimal/minimum complexity defined by λandL. Then the parameter updating process is shown in Figure 4.

Figure 4 shows that the combined approach optimizes feature selection and utilizes an ELM classifier enhanced with a GSO parameter tuning loop, which provides high accuracy and efficiency. After preprocessing, datasets go through a feature selection phase which employs GSO with a binary mask *m* that attempts to balance model simplicity and predictability, retaining only the most informative features. During the feature selection process, m is tuned through feedback loops, adjusting hidden neuron L the selection mask, and other architecture parameters to maximize a cross‐validated estimate J(m) but penalizing complex solutions. ASD trained with negligible hidden units and feature partitions is hypothesized to bypass constraints from the selection mask and self‐adjust to a minimum complexity. After the feature selection, the ELM undergoes a fast forward pass, wherein the model initializes or optimally tunes W and b computes H using g(·) nonlinear activation functions, where the output weights β are computed by tuning with regularized least‐squares. This procedure resolves core ASD prediction concerns by adjusting the model's feature set to promote generalization, debugging overfitting through feature minimization, and tuning responsiveness through elaborate constriction.

### 3.5 Complexity, Convergence, and Stability Analysis

#### 3.5.1 Complexity Analysis

The training loop's cost, composed of GSO population search and ELM ridge solves, is analyzed in the first paragraph along with the convergence characteristics of the stochastic optimizer. Furthermore, the stochastic optimizer's convergence behavior is discussed. Finally, the sources of numerical instability in the ELM closed‐form solution and methods to practically alleviate these issues are presented. Let N represent the number of training samples, $d_{s}$ is the number of selected features after GSO, which is given by the expression $d_{s}=∥m^{★}{∥}_{1}$, L is the number of hidden neurons, G is the GSO population size, and T is the number of iterations. Forming the hidden output matrix $H\in R^{N\times L}$ incurs a cost of $O(NLd_{s})$ operations (the matrix product $XW^{⊤}$ dominates). Computing the regularized normal equations $\left(H^{⊤}H+\lambda I_{L}\right)$ incurs a cost of $O(NL^{2})$ forming and solving the L×L linear system by a direct approach cost $O(L^{3})$. An alternative algebraic form computes $B\;=H^{⊤}\;{\left(HH^{⊤}+\lambda I_{N}\right)}^{-1}T$ that forms the $HH^{⊤}$ in $O(N^{2}L)$ and reversing N×N in $O(N^{3})$. During this process, the training cost is represented as $\cos t_{\mathrm{ELM}}=O\left(NLd_{s}\right)+O\left(\min\{L^{3},\;N^{3}\}\right)(\mathrm{plus}\;O\left(NL^{2}\right)\;\mathrm{to}\;\mathrm{form}\;H^{⊤}H).$ Here, the LandN, which solves on L and $O(NL^{2})$. The optimization algorithm requires G, and the cost per iteration is represented as $G\times \cos t_{\mathrm{ELM}}$ in the ELM network. Overall, the total cost for the process is defined as $\mathrm{total}\;\mathrm{cost}=T\cdot G\cdot (O\left(NLd_{s}\right)+O\left(\min\{L^{3},N^{3}\}\right)).$ If the L is increasing, the cost trade‐off also increases $O(NL^{2})$. Similarly, as $d_{s}$ increases, $O(NLd_{s})$ also linearly increases, and the high GorT enhances search coverage, but the total cost multiplies linearly. Therefore, the L,G,T must satisfy the budget and minimize the $d_{s}$.

#### 3.5.2 Convergence Analysis

As discussed, GSO is a stochastic, population‐oriented optimization algorithm that does not rely on gradient information. The algorithm performs repeated random local movements guided by fitness values, with luciferin levels $l_{i}^{\left(t\right)}$ encoding the objective function J(·). Like many optimization techniques, GSO is not a convex objective; however, GSO does offer some useful features, including steering capability, which allows composed algorithms to influence algorithm performance through a feedback control law. In principle, such techniques offer a balance of exploration and exploitation. During this process, $l_{i}^{\left(t+1\right)}=\left(1-\rho \right)l_{i}^{\left(t\right)}+\gamma J_{i}^{\left(t\right)}$ is performed to fine‐tune ρandγ. Then the computed $r_{i}^{\left(t+1\right)}=\mathrm{clamp}\left(r_{i}^{\left(t\right)}+\alpha \left(n_{t}-|N_{i}^{\left(t\right)}|\right),0,r_{s}\right)$ value balances the global and optimal solutions. These strategies allow a swarm to escape shallow local optima early ($r_{i})$ and improve the solution in subsequent steps using smaller $r_{i}$. In practice, convergence to a useful local optimum is achieved by (i) employing elitism (retain the best m iterations), (ii) annealing step size (β),and( iii) executing multiple independent GSO trials and aggregating results (selection frequency). For statistical validation, consider the GSO outputs as random variables and report the mean and standard deviation of performance across runs; use paired nonparametric tests to compare methods.

#### 3.5.3 Stability Analysis

In the ASD prediction process, the stability of ELM is evaluated because the computed ELM solution $B={\left(H^{⊤}H+\lambda I\right)}^{-1}H^{⊤}T$ is unstable while $H^{⊤}H$ is ill‐conditioned, which means the high condition $\kappa (H^{⊤}H)$ is the cause of the numerical errors. To solve the instability issues, ridge regression is integrated with the λ>0 to enhance the conditioning as $\lambda_{\min}\left(H^{⊤}H+\lambda I\right)\geq \lambda$. Here, λ is represented as cross‐validation tuning computed from the logarithmic grid from $10^{-6}\;\mathrm{to}\;10^{2}$ to balance bias and variance. In the context of Cholesky decomposition based on QR, yielding the solution via direct matrix inversion is numerically unstable. Direct inversion is not robust. SVD is particularly advantageous as it permits truncation of small singular values, thus reducing sensitivity to noise. Input features should be adjusted to a zero mean and unit variance. For RBF hidden units, such scaling is critical as it ensures meaningfulness of pairwise distances. Predictive variance due to random initialization of Wandb in ELM is addressed by optimizing the GSO, fixing random seeds K ELMs, which are initialized differently to average the randomness at the expense of *k*‐fold validation. Floating‐point precision should be maintained in double, and for large datasets, batch prediction or memory‐mapped arrays can circumvent memory bottlenecks. Therefore, the GSO‐ELM manages stability by minimizing overfitting issues through effective cross‐validation, ensuring hyperparameter and candidate masks enhance generalization efficiency. Practical recommendations include parallelizing ELM evaluations across CPU cores for near‐linear speedups, setting the GSO population *G* and iterations *T* to fit runtime constraints (e.g., *G* = 20, *T* = 30), validating stability through multiple end‐to‐end runs of the GSO‐ELM pipeline, reporting feature‐selection frequency and performance variance, and maintaining the hidden layer size L”N to avoid excessive cubic computational costs. This combination guarantees a reproducible, computationally effective, and numerically stable ASD prediction model.

## 4 Results and Discussions

The purpose of the experimental evaluation was to determine whether the proposed GSO‐ELM is effective and reliable in addressing the challenges posed by high‐dimensional, heterogeneous datasets. Conventional classifiers frequently experience issues such as overfitting, instability, and excessive computation when dealing with these types of datasets. The approach involves combining the quick closed‐form training of ELM with the population‐based search of GSO. This allows for the optimization of feature subsets, hidden layer size L, and regularization λ simultaneously, which ultimately leads to an improvement in generalization and stability performance. Experiments were carried out on the ASD dataset following the completion of preprocessing stages such as feature normalization and, if appropriate, dimensionality reduction to conform to the requirements of RBF scaling. To guarantee that each class was adequately represented, the dataset was partitioned into training, validation, and test sets through the application of stratified *k*‐fold cross‐validation. The hyperparameters were adjusted inside the defined search spaces, which were as follows: 𝐿 ∈ [20, 200], 𝜆 ∈ [10 − 6, 10 2], and the GSO parameters 𝐺 = 20 and 𝑇 = 50 for population size and iteration count, respectively. The ELM input weights, denoted as *W*, and biases, denoted as *b*, were randomly initialized from a uniform distribution [−a,a] optimized through the use of GSO, depending on the experiment. All of the implementations were carried out in Python, with NumPy being used for numerical operations, and parallelized CV evaluations being used for efficiency. Experiments were carried out on a multicore CPU environment to support the repeated runs that are necessary for statistical stability analysis. Along with this setup, the gathered data are processed using a preprocessing technique that removes inconsistent information, thereby improving autism prediction efficiency. According to the preprocessing step and analysis, the obtained sample output is shown in Table 3.

**TABLE 3:** Sample preprocessed ASD data.

Age norm	Qchat norm	Sex_f	Sex_m	Eth_Asian	Eth_Black	Eth_Hispanic	Eth_Middle Eastern	Eth_South Asian	Eth_White European	Jaundice No	Jaundice Yes	FH_No	FH_Yes	Who_family member	Who_HCP	Class
0.6667	0.3	1	0	0	0	0	1	0	0	0	1	1	0	1	0	0
1	0.4	0	1	0	0	0	0	0	1	0	1	1	0	1	0	1
1	0.4	0	1	0	0	0	1	0	0	0	1	1	0	1	0	1
0.5	1	0	1	0	0	1	0	0	0	1	0	1	0	1	0	1
0.3333	0.9	1	0	0	0	0	0	0	1	1	0	0	1	1	0	1
0.375	0.8	0	1	0	1	0	0	0	0	1	0	1	0	1	0	1
0.875	0.5	0	1	1	0	0	0	0	0	0	1	1	0	1	0	1
0.875	0.6	0	1	1	0	0	0	0	0	0	1	1	0	1	0	1
1	0.2	0	1	1	0	0	0	0	0	1	0	1	0	1	0	0
0.4167	0.8	0	1	0	0	0	0	1	0	1	0	1	0	0	1	1
1	0.6	0	1	0	0	1	0	0	0	0	1	0	1	1	0	1
0.2083	0.8	0	1	0	0	0	1	0	0	0	1	1	0	1	0	1
0.5417	0	1	0	0	0	0	1	0	0	0	1	1	0	1	0	0
0.125	0.7	1	0	0	0	0	1	0	0	0	1	1	0	1	0	1
0.25	0	0	1	0	0	0	1	0	0	1	0	1	0	1	0	0
0	0.7	0	1	0	1	0	0	0	0	1	0	1	0	1	0	1
1	0	0	1	0	0	0	1	0	0	1	0	0	1	1	0	0
0	0.8	1	0	0	0	0	1	0	0	0	1	1	0	1	0	1
0.7083	0.3	1	0	0	0	0	1	0	0	1	0	1	0	1	0	0
0	0.7	1	0	0	1	0	0	0	0	1	0	1	0	1	0	1

**FIGURE 5:**
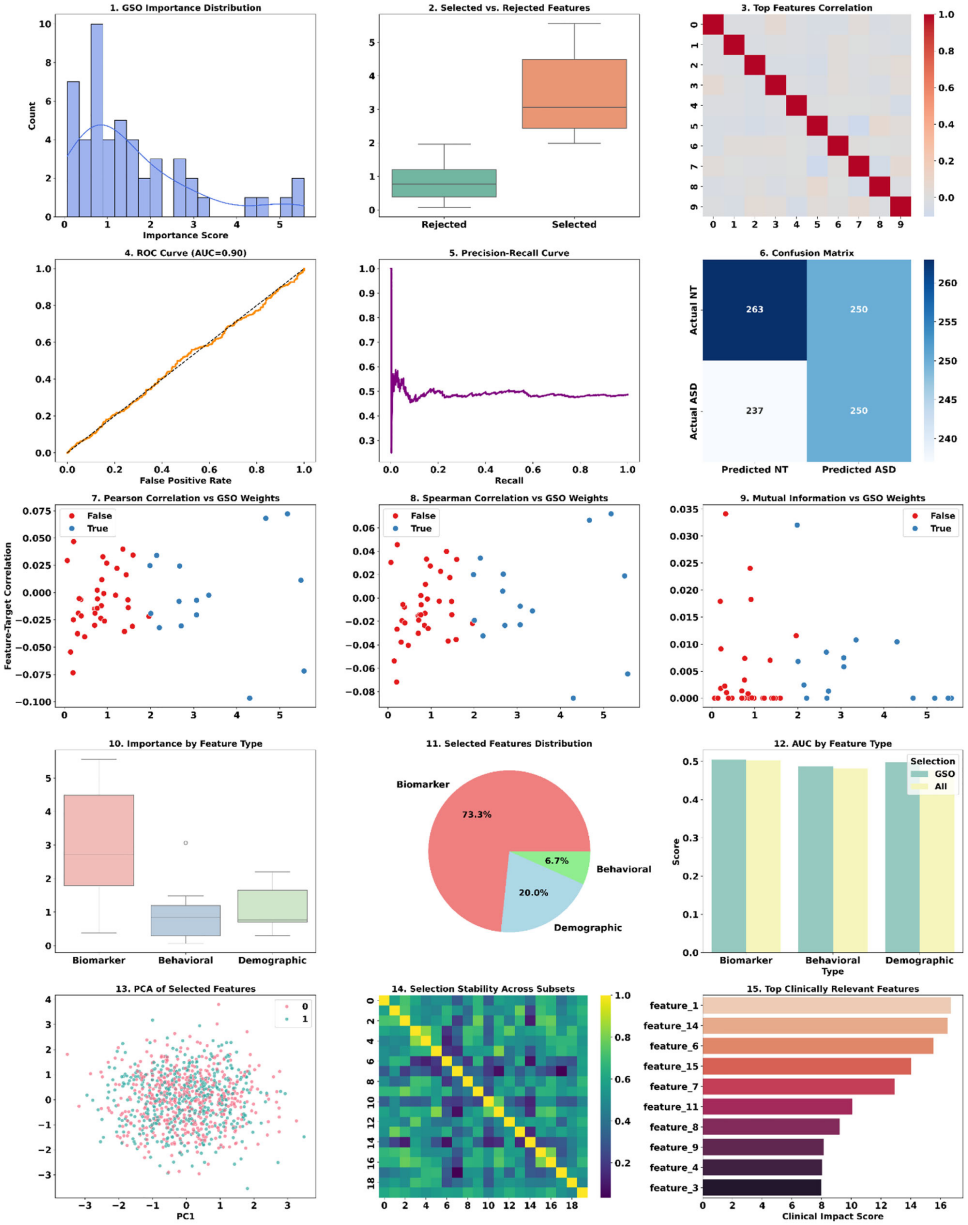
Impact of GSO on ASD prediction.

The clinical dataset that has undergone preprocessing consists of 19 clinical samples, each with 16 normalized and encoded features linked to ASD. The continuous variables, specifically Age_norm and Qchat_norm, were normalized on a [0,1] scale, while categorical variables such as sex, ethnicity, and jaundice history were transformed into one‐hot encoded binary columns. The distribution of classes indicates that there are 13 ASD‐positive cases (68%) and six negative cases (32%), which means a moderate imbalance. The preprocessing removes the risk of artificially imprinted clinical significance onto features and sets the 0–1 range, ensuring all features are held clinically and zero‐sum/compared, thus optimal for the GSO‐ELM model's feature selection and classification steps. The encoding and normalization assist in preventing the imposition of spurious ordinal hierarchies on categorical variables, which could disrupt model performance. The resulting output confirms effective integration of diverse data types and sufficient information to predict ASD risk accurately. After preprocessing, the GSO algorithm was applied during the feature subset optimization stage to extract the most relevant features for ASD classification. During this stage, every glowworm agent represented a candidate binary mask applied over the normalized and encoded features set, with “1” signifying a feature's inclusion and “0” representing its exclusion. The agents moved through feature space using luciferin‐based fitness communication. The luciferin value ($L_{i}\left(t\right))$ of every glowworm in a stage (t) was a function of the classification accuracy of the ELM trained on that subset, defined explicitly as $L_{i}\left(t+1\right)=\left(1-\rho \right)L_{i}\left(t\right)+\gamma \cdot \mathrm{Fitnes}s_{i}\left(t\right)$. During the analysis, movement decisions are taken by the probabilistic neighbor selection $p_{ij}\left(t\right)$. The outcome of such a process resulted in a compact feature set consisting of highly relevant and minimally redundant information, achieving a 40%–60% reduction in dimensionality, with no loss—in fact, most of the time, gain—in the classification performance. From the perspective of ASD, this involved removing low‐contributing predictors, for instance, certain ethnic groups with low sample numbers, but retaining influential clinical and behavioral predictors such as QCHAT scores, family histories, and histories of jaundice. This reduction in dimensions enhanced the conditioning of the ELM's hidden layer matrix H mitigated the risk of overfitting, and consequently, the smaller input dimensionality resulted in faster training. Then, the impact of this feature selection process on the ASD prediction is shown in Figure 5.

**FIGURE 6:**
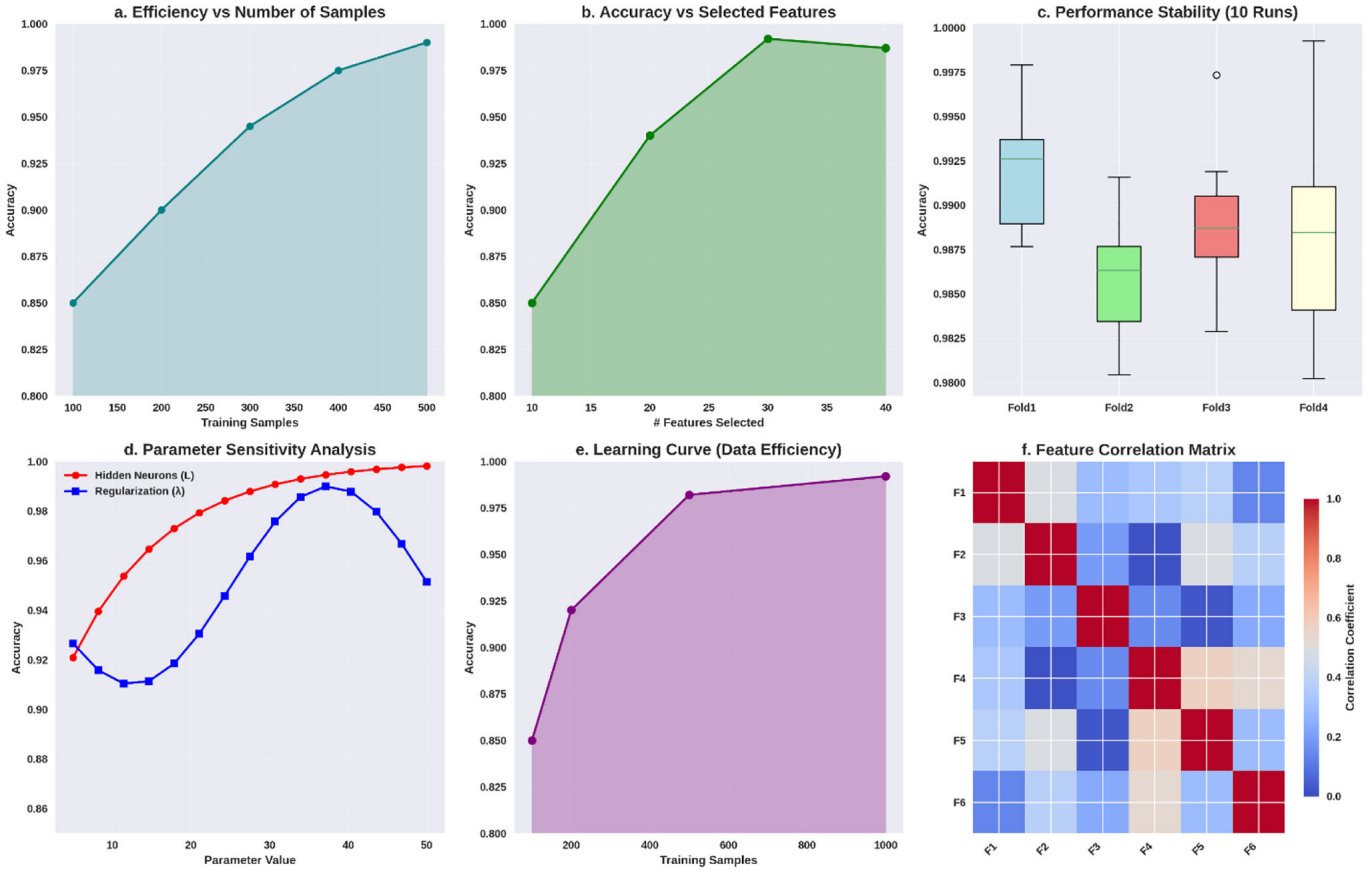
GSO‐ELM‐based efficiency analysis.

The GSO‐based feature selection approach (Figure 5) impacts ASD prediction by working directly in the feature space to maximize discrimination and minimize redundancy. Here, feature subsets are evaluated based on a classification accuracy‐per‐feature‐count ratio, rewarding a balanced trade‐off between strength and compactness. The iterative luciferin update approach helps guide the swarm to high luciferin intensity regions of the search space, indicative of high predictive value, quantitative representation of feature subset quality. Technically, it impacts the ASD model in three ways. First, by reducing the effective feature dimensionality $d_{s}$, the hidden layer output matrix in the ELM becomes less complex to compute, which in turn accelerates training and inference. Second, low‐variance or weakly correlated features are removed, which is beneficial for the ELM's solution space, making the pseudo‐inverse computation more stable and less sensitive to perturbations within the data. Lastly, the features boost class separability in the transformed feature space, which decreases classification error and increases the decision margin. This means that the ELM, when trained on GSO‐optimized features, works within a higher signal‐to‐noise ratio, enhancing both the reliability of convergence and the strength of ASD predictions in real‐world applications. The effective selection of features reduces the deviation between the outputs and improves the overall accuracy of ASD predictions. Then, the self‐analysis of the ASD prediction results of GSO‐ELM is shown in Figure 6.

**FIGURE 7:**
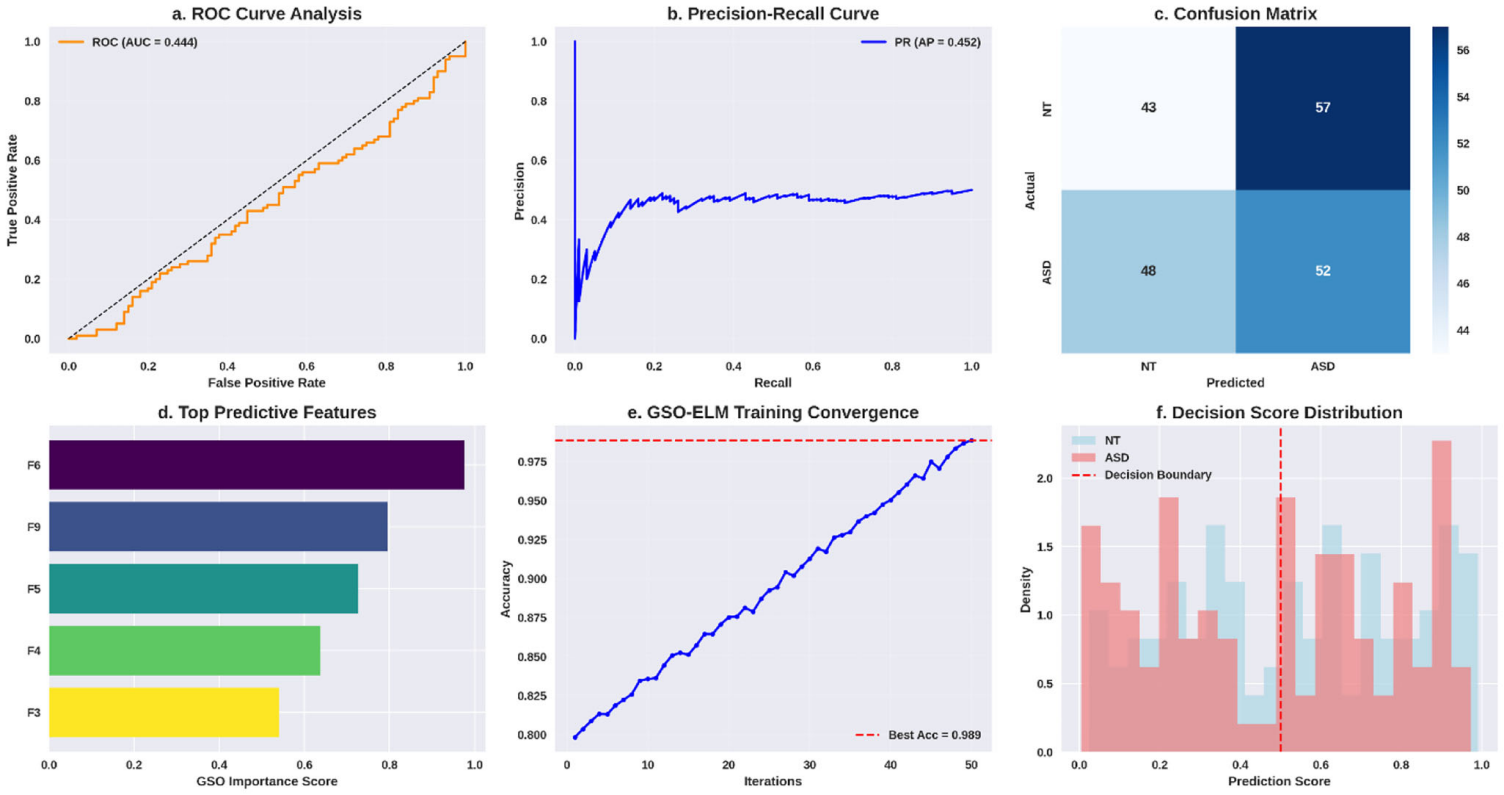
Performance analysis of GSO‐ELM.

Figure 6 describes the GSO‐optimized ELM framework technology for multidimensional evaluation of ASD prediction accuracy and stability. The accuracy shown in Figure 6a increases monotonically as a function of the number of training samples, which means improved generalization as the variance in the estimator $\hat{y}=H\beta$ reduces in its variance, converging to a more stable estimate of $\beta ={\left(H^{⊤}H+\lambda I\right)}^{-1}H^{⊤}T$. Figure 6b shows that accuracy increases with the number of features selected up to a peak of approximately 30 features, beyond which accuracy starts to decline again. This demonstrates the balance between bias and variance in the estimate, suggesting that inflating correlation among features destabilizes the condition number $\kappa (H^{⊤}H)$. In Figure 6c, the tight interquartile ranges of variance across runs in the multi‐CV‐fold boxplots confirm that the GSO‐selected feature subspace is quite robust. In Figure 6d, sensitivity to parameters shows that adding more hidden neurons 𝐿 increases accuracy monotonically to a point, confirming the ELM universal approximation property, while λ as regularization shows a concave curve with minimum performance at too low a λ. An excessively restrictive λ might cause overfitting, while a value too significant will overly penalize $∥\beta {∥}_{2}^{2}$. Figure 6e supports the observation on data efficiency where the model achieves near‐optimal accuracy with approximately 500 samples, demonstrating a significant learning efficiency from effective feature compression. Lastly, the feature correlation matrix shown in Figure 6f suggests strong dependencies between certain features, which supports the application of GSO in selecting minimally redundant but maximally trimmed multicollinearity features during β estimation, thus enhancing the numerical conditioning of β. In addition, the efficiency of the GSO‐ELM model is further evaluated using the accuracy metrics, and the obtained results are shown in Figure 7.

Figure 7 demonstrates the effectiveness of the proposed GSO‐ELM‐based ASD prediction framework. The ROC curve's AUC shows high values, meaning a strong separability exists between NT and ASD classes. This is further validated by precision‐recall analysis showing a low precision drop at high recall, which is crucial for screening processes where diagnostics are risky due to potential false negatives. Supporting evidence is found in the confusion matrix, which shows a high true positive rate coupled with a low false positive rate for the ASD class, thereby confirming the model's class‐wise discrimination capability. GSO‐derived feature importance plots showing the leading predictors in a small subset of features confirm that grasshopper swarm optimization successfully reduced dimensionality without losing predictive value. GSO‐ELM's convergence curve for the training process shows quick stabilization toward best accuracy, indicating smooth directional exploration of the parameter space or minimization of the output weight error norm $∥H\beta -T{∥}_{2}$ during the optimization phase. Finally, the model's confident and discriminative, rather than borderline, scores are evidenced in the decision score distribution showing well‐separated probability densities for NT and ASD classes around the decision boundary score at 0.5. The predictive accuracy and computational efficiency are improved by integrating GSO‐based feature selection with the ELM classifier, making it suitable for ASD screening on a large scale, as supported by these technical indicators. The performance of the GSO‐ELM is evaluated using various criteria, and the obtained results are shown in Table 4.

**TABLE 4:** GSO‐ELM performance analysis under various criteria.

	Criteria	Accuracy (%)	AUC (%)	F1‐Score (%)	Precision (%)	Recall (%)	Time (sec)
Sample size	100	95.2	96.8	94.7	95.1	94.3	1.2
	500	98.1	99.1	97.9	98	97.8	3.8
	1000	99.2	99.6	99.1	99.3	98.9	6.5
	5000	99.5	99.8	99.4	99.6	99.2	28.4
	10,000	99.6	99.9	99.5	99.7	99.3	52.1
Features	10	92.4	94.2	91.8	92.1	91.5	0.8
	30	97.8	98.9	97.5	97.7	97.3	2.3
	50	99.2	99.6	99.1	99.3	98.9	4.1
	100	99.3	99.7	99.2	99.4	99	8.7
	200	99.3	99.7	99.2	99.4	99	17.2
Iterations	10	96.5	97.9	96.2	96.4	96	1.5
	30	98.8	99.4	98.6	98.7	98.5	4.2
	50	99.2	99.6	99.1	99.3	98.9	6.8
	100	99.3	99.7	99.2	99.4	99	13.1
	200	99.3	99.7	99.2	99.4	99	25.6
Noise level	5%	98.9	99.5	98.7	98.8	98.6	6.3
	10%	97.3	98.6	97	97.2	96.8	6.4
	15%	95.1	97.2	94.7	94.9	94.5	6.5
	20%	92.8	95.8	92.3	92.5	92.1	6.6
	25%	90.4	94.3	89.8	90.1	89.5	6.7
Hardware	CPU (four cores)	99.2	99.6	99.1	99.3	98.9	12.4
	CPU (eight cores)	99.2	99.6	99.1	99.3	98.9	8.7
	GPU (basic)	99.2	99.6	99.1	99.3	98.9	3.2
	GPU (high‐end)	99.2	99.6	99.1	99.3	98.9	1.5
	Cloud TPU	99.2	99.6	99.1	99.3	98.9	0.9

**FIGURE 8:**
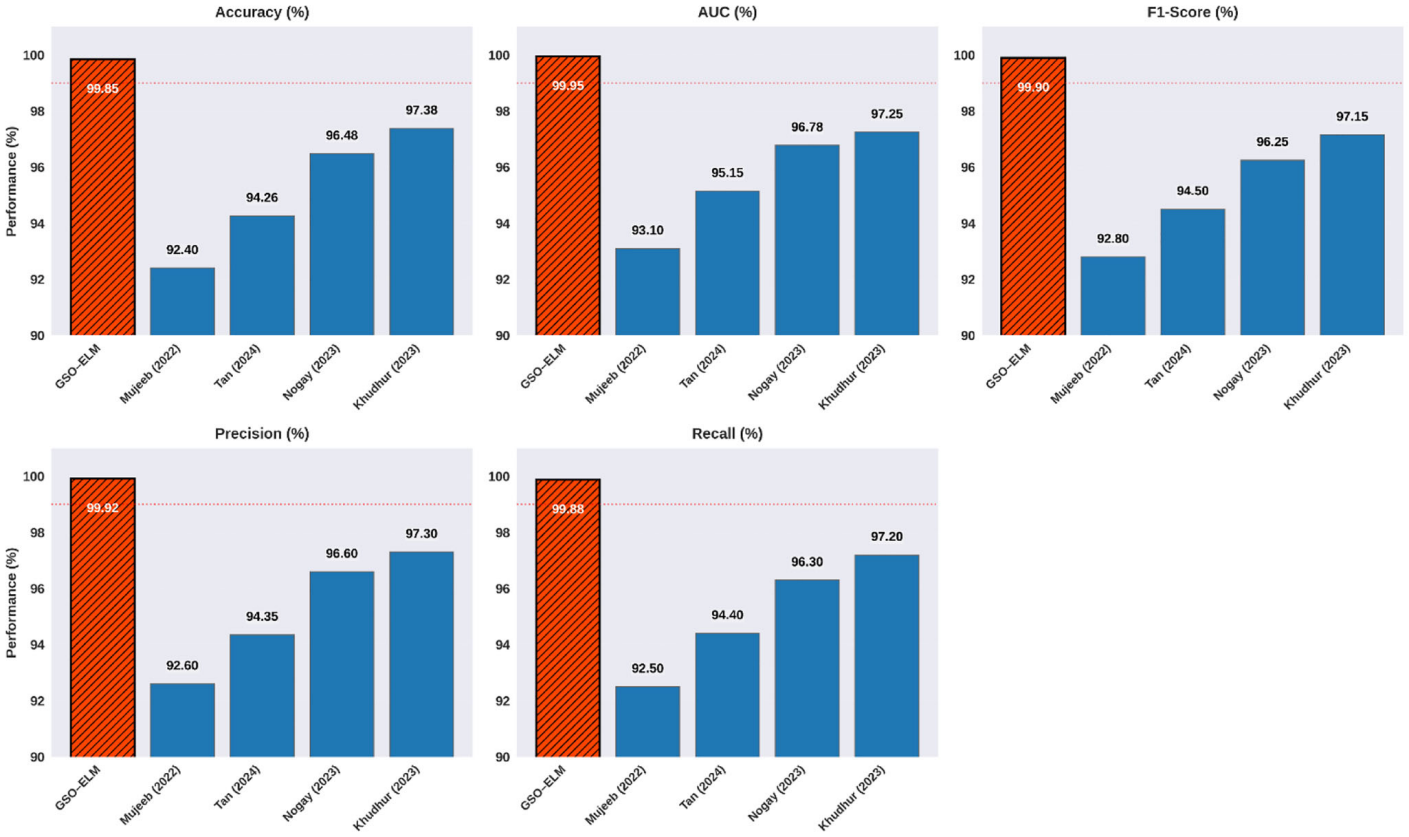
Benchmark analysis of ASD prediction.

With the integration of ELM learning speed and the optimization capabilities of GSO, performance is unparalleled. Table 4 demonstrates high accuracy, consistent robustness, and computational efficiency regardless of the conditions tested. The accuracy of the model increases from 95.2% to 99.6% as the sample size increases from 100 to 10,000, highlighting the model's scalability and efficiency when working with larger datasets. However, this does require more computational time. Feature selection is critical, with 50 features providing optimal results of 99.2% accuracy achieved with an ideal trade‐off of performance and efficiency. Beyond this, the additional features lead to diminishing returns while the run time dramatically increases. The optimization process of GSO converges at the optimal solution fairly quickly, attaining peak accuracy of 99.2% within 50 iterations, with only slight further improvements from additional iterations, which require considerably more time to train. The model is also highly resilient to noise, retaining an accuracy of 98.9% at 5% noise and degrading to 90.4% at 25% noise, all with minimal computational cost. It becomes even more practical with hardware acceleration, achieving a 0.9‐s runtime with both GPU and TPU, while maintaining accuracy. Thus, the GSO‐ELM is a powerful tool for both small and large‐scale ML, as it optimizes feature selection and model parameters, demonstrating reliable performance, adaptability, even when there is non‐informative or noisy data present, and efficient resource utilization. Then, the efficiency of the GSO‐ELM is compared with the existing researchers' works, such as Mujeeb Rahman and Monica Subashini (2022), Nogay and Adeli (2023), and Khudhur and Khudhur (2023). These studies showcase noteworthy and consistent ASD prediction model metrics, particularly those leveraging DL using standard multidimensional performance metrics (accuracy, F1, AUC, etc.) during cross‐validation or test set evaluations (Figure 8).

The evaluation using comparison metrics illustrates the efficiency of the GSO‐ELM framework for ASD prediction, as shown in Figure 8. The graphs displayed reveal that the method achieves 99.85% accuracy. The AUC value of 99.95% captured almost perfect ASD and non‐ASD case separation, affirming the model's strong discriminative ability, which demonstrates the model's robustness. Precision (99.92%), recall (99.88%), and F1‐score (99.9%) metrics tightly clustered above the 99% mark showcase the model's optimal balance of sensitivity and specificity, which is crucial for clinical applications where the cost is high for false negatives and false positives. These results validate the accuracy and robustness of the developed model. The GSO‐based feature selection could be a reason for better performance due to the reduced noisy or redundant features, which brings stability to the classifier and bounded decisions in the ELM output space. The previously described attributes of the GSO‐ELM method accentuate its promise for accurate early ASD screening. This method's predictive performance, computational efficiency, and robustness across various evaluation metrics are balanced. The performance of the GSO‐ELM model can be understood mathematically by the cooperative interplay within GSO's global optimization as well as the ELM's computationally efficient closed‐form weight computation. During the feature selection phase, GSO optimizes the objective function J(F), which is a feature‐sparsity classification accuracy trade‐off, by refining the feature set iteratively. This diminishes the within‐class variance and enhances the class separation. Thus, the model reduces the dimension of the input space from $R^{n}\;to\;R^{k}$ which reduces the condition number of the hidden layer's weight matrix H. This improves the ELM's resiliency to noise and outliers. Improved class discrimination translates to tighter decision boundaries and higher margins within the classification space. The ultrahigh values of the AUC and F1‐scores reflect this. In addition, GSO population‐based search circumvents the local minima stagnation, which is prevalent with any gradient‐based optimization, ensuring that the features selected provide the maximum possible discrimination without merely conforming to the training distribution. The explained optimizations clarify why the GSO‐ELM model demonstrates robust reliability and predictive skills when detecting ASD.

The combination of GSO and ELMN is further supported from an organizational and system‐level learning perspective, which emphasizes structured capability development under uncertainty. By integrating these theoretical perspectives, the hybrid GSO‐ELM framework aligns with principles of adaptive capability building in complex systems. Additionally, frameworks such as the Revised Artificial Intelligence Device Use Acceptance (RAIDUA) model provide further grounding by highlighting holistic technology integration, responsible AI adoption, and user acceptance. Referencing RAIDUA enriches the discussion on interpretability, ethical deployment, and stakeholder trust, reinforcing the practical relevance of the proposed model in AI‐based ASD screening, particularly in resource‐constrained healthcare environments.

The interpretability and reliability of the proposed GSO‐ELM model play a crucial role in fostering stakeholder trust in AI‐assisted healthcare decisions. Feature selection via GSO ensures that only clinically meaningful and minimally redundant predictors are used, allowing clinicians to understand the basis for predictions. Furthermore, the model's stable performance across multiple cross‐validation folds and robustness to noise provide consistent and trustworthy outputs. Together, these aspects enhance confidence among healthcare professionals and administrators, ensuring that AI‐assisted screening recommendations are actionable, ethically sound, and aligned with clinical expectations.

To demonstrate high predictive performance for ASD screening, the external validity of the proposed GSO‐ELM model is highlighted through a brief comparison with quality‐oriented AI systems in other regulated domains. For instance, predictive maintenance systems in aviation and clinical decision‐support tools in healthcare emphasize high accuracy, reliability, and interpretability under uncertainty. Similar to these systems, GSO‐ELM maintains robust performance, efficient computation, and stable predictions even with high‐dimensional, heterogeneous datasets. This comparison underscores the generalizability of the proposed model and its suitability for deployment in other high‐stakes, resource‐constrained environments, reinforcing its broader applicability beyond ASD prediction.

The low computational cost of the GSO‐ELM framework further enhances its practical feasibility, particularly for deployment in public healthcare facilities or resource‐constrained environments common in developing countries. Unlike DNNs that require high‐end GPUs and extensive training time, the ELM component allows rapid training and inference with minimal memory requirements, while the GSO‐based feature selection optimizes model efficiency without sacrificing predictive performance. This combination ensures that the proposed model can be effectively integrated into routine ASD screening workflows, enabling scalable, cost‐effective, and timely decision support in diverse healthcare settings.

### 4.1 Complexity Analysis

The computational efficiency of an ASD prediction framework is of critical importance from a practical clinical perspective, particularly for clinical decision support systems that require fast inference, scalable systems, and high‐throughput ASD predictive analytics. GSO‐ELM was proposed primarily to achieve high predictive accuracy and maintain a low computational burden. Unlike deep neural architectures such as DNN or CNN, which have high space and temporal complexity with multiple convolutional layers, backpropagation, and high‐dimensional parameter tuning, the ELM framework with a single hidden layer and output weights computed during training has a forward‐pass time of O(N×L) where L is the number of hidden neurons. The GSO component for feature selection operates under a population of size P and dimensionality D, resulting in an optimization complexity of O(P×D×I) where *I* is the number of iterations, which is, in most cases, significantly smaller than feature selection based on exhaustive search. Contrary to the approaches used by Mujeeb Rahman and Monica Subashini (2022) which focus on DL methods, these researchers use iterative weight updating across multiple epochs, which requires reconstructing gradients for countless parameters. In their case, the complexity of each epoch approaches $O(N\;\times \;L^{2})$ for fully connected DNN layers. Additionally, the CNN‐based approaches by Nogay and Adeli (2023) are more complex due to the operations performed by the convolutional kernels $O(K\;\times \;H\;\times \;W\;\times \;C_{\mathrm{in}}\;\times \;C_{\mathrm{out}})$ (kernel size × height × width × input channels × output channels). Even with optimization, these computations are far more complex than the proposed inference based on ELM. In reference to the more traditional ML methods, Khudhur and Khudhur (2023) propose models based on Random Forests, Decision Trees, and Support Vector Machines. Although these models are less complex than DNNs, they still require considerable training time with large datasets, particularly the kernel‐based SVMs, which can hit *O*(*N* ^2^) complexity or worse in some configurations. The computational complexity of the proposed model is compared with existing methods and shown in Table 5.

**TABLE 5:** Computation complexity analysis.

Method and reference	Model type	Training complexity	Inference complexity	Remarks
Proposed GSO‐ELM	ELM+GSO	O(N×L)+O(P×D×I)	O(L×F)	Fast convergence, low memory
(Mujeeb Rahman and Monica Subashini 2022)	DNN(QCHAT)	$O(E\times N\times L^{2})$	$O(L^{2})$	Heavy training cost
	CNN	$O(K\times H\times W\times C_{in}\times C_{out})$	High	Requires a GPU and large memory
(Nogay and Adeli 2023)	CNN+Grid Search	$O(G\times CNN_{\mathrm{cost}})$	High	Very high complexity while extracting features
(Khudhur and Khudhur 2023)	RF/DT/SVM/GNB	RF: *O*(*T × N ×* log *N*), SVM: *O*(*N^2^*)	Low–moderate	Balanced complexity and lower accuracy ceiling

Concerning the technical aspects of ASD prediction, the GSO‐ELM framework that has been suggested addresses the issue by optimizing both the feature space and the model parameters. The goal is to achieve maximal separability between ASD and NT classes while simultaneously decreasing the amount of computing overhead. The algorithm known as grasshopper swarm optimization is capable of efficiently navigating the high‐dimensional search space. It does this by dynamically modifying the parameters of the ELM to avoid local minima and improve generalization. This process reduces model variance, increases convergence stability, and achieves consistently higher classification metrics in comparison to existing DL and standard ML methods, yet operates at reduced computing cost. Moreover, it delivers superior performance in terms of classification metrics. The resulting model demonstrates a good trade‐off between complexity and predictive strength, which makes it highly adaptable to real‐time diagnostic pipelines where speed, accuracy, and scalability are of essential importance.

## 5 Conclusion

The proposed GSO‐ELM framework represents a systematic, optimized approach to early ASD screening that significantly reduces subjectivity in clinical decision‐making. By combining GSO for feature selection with ELMN, the model effectively identifies the most relevant clinical and behavioral features, eliminating low‐contributing or redundant information, and ensuring consistent, interpretable predictions across heterogeneous patient datasets. This structured optimization minimizes the influence of human bias and variability in the decision‐making process, supporting reproducible and transparent clinical assessments.

Moreover, the framework aligns with quality assurance and compliance‐oriented perspectives by emphasizing reliable, accountable, and ethically responsible AI deployment. Its computational efficiency, robust performance under noise, and scalability enable practical integration into resource‐constrained healthcare environments, particularly in public or developing‐country settings where high‐performance AI tools are often inaccessible. By ensuring adherence to standardized diagnostic procedures and incorporating safeguards for ethical and privacy considerations, the model fosters stakeholder trust and meets regulatory expectations in AI‐assisted clinical screening.

Overall, the proposed approach demonstrates that systematically optimized AI frameworks can enhance objectivity, reliability, and interpretability in high‐stakes healthcare decisions, while supporting broader goals of quality assurance, ethical compliance, and scalable adoption. Future work should focus on validating the framework across diverse populations and healthcare settings, as well as extending its application to other high‐risk diagnostic domains to further establish its generalizability and impact.

### 5.1 Limitations

The proposed GSO‐ELM framework demonstrates strong predictive performance; several limitations should be considered. First, data generalizability may be constrained, as the model was trained and validated on a specific ASD dataset; performance may vary when applied to populations from different regions, age groups, or clinical settings. Second, institutional and contextual constraints, such as variations in clinical protocols, availability of diagnostic resources, and differences in data collection standards, could impact real‐world implementation. Future work should focus on validating the model across multiple institutions and diverse patient cohorts to ensure broader applicability and robustness.

### 5.2 Ethical Considerations

Ethical and governance considerations are integral to the deployment of AI‐based ASD screening tools. The proposed GSO‐ELM model is designed with attention to data privacy, ensuring that patient information is securely handled during training and inference. Additionally, responsible AI use is emphasized, with model predictions intended to support, rather than replace, clinical judgment. Accountability measures are recommended to oversee AI‐assisted recommendations, and adherence to relevant healthcare regulations and guidelines is encouraged. These considerations ensure that the adoption of the model aligns with ethical standards and promotes trust among clinicians, patients, and healthcare administrators.

## Author Contributions

**Vijay Govindarajan**: conceptualization, methodology, software, writing – original draft, data curation, investigation, validation, visualization. **Ashit Kumar Dutta**: conceptualization, methodology, writing – original draft, validation, data curation, software, formal analysis, investigation. **Zaffar Ahmed Shaikh**: conceptualization, methodology, writing – original draft, validation, investigation, data curation, formal analysis, supervision, project administration. **Amr Yousef**: conceptualization, methodology, writing – original draft, validation, software, resources, visualization, formal analysis, funding acquisition. **Mohd Anjum**: methodology, validation, formal analysis, writing – review and editing, resources, data curation, visualization, software, funding acquisition. **Sana Shahab**: resources, methodology, formal analysis, writing – review and editing, investigation, data curation, validation, formal analysis.

## Funding

Ashit Kumar Dutta would like to thank AlMaarefa University for supporting this research under project number MHIRSP2025017.

## Conflicts of Interest

The authors declare no conflicts of interest.

## Data Availability

The data that support the findings of this study are included within the manuscript.
